# Combination of Withaferin-A and CAPE Provides Superior Anticancer Potency: Bioinformatics and Experimental Evidence to Their Molecular Targets and Mechanism of Action

**DOI:** 10.3390/cancers12051160

**Published:** 2020-05-05

**Authors:** Anissa Nofita Sari, Priyanshu Bhargava, Jaspreet Kaur Dhanjal, Jayarani F. Putri, Navaneethan Radhakrishnan, Seyad Shefrin, Yoshiyuki Ishida, Keiji Terao, Durai Sundar, Sunil C. Kaul, Renu Wadhwa

**Affiliations:** 1 DAILAB, DBT-AIST International Center for Translational and Environmental Research (DAICENTER), National Institute of Advanced Industrial Science & Technology (AIST), Tsukuba 305-8565, Japan; sari-anissa@aist.go.jp (A.N.S.); bhargava.priyanshu@aist.go.jp (P.B.); jaspreetk.dhanjal@aist.go.jp (J.K.D.); jayaranidna@gmail.com (J.F.P.); 2 School of Integrative and Global Majors, University of Tsukuba, Tsukuba 305-8577, Japan; 3 DAILAB, Department of Biochemical Engineering and Biotechnology, Indian Institute of Technology (IIT) Delhi, Hauz Khas, New Delhi 110 016, India; bez178438@dbeb.iitd.ac.in (N.R.); bez188440@dbeb.iitd.ac.in (S.S.); sundar@dbeb.iitd.ac.in (D.S.); 4 CycloChem Co. Ltd., 7-4-5 Minatojima-Minamimachi, Chuo-ku, Kobe 650-0047, Japan; yoshiyuki.ishida@cyclochem.com (Y.I.); keiji.terao@cyclochem.com (K.T.)

**Keywords:** Ashwagandha, Withaferin A, Honeybee propolis, CAPE, Cancer, Mortalin, p53, PARP1, Regulation

## Abstract

We have earlier reported anticancer activity in Withaferin A (Wi-A), a withanolide derived from Ashwagandha (*Withania somnifera*) and caffeic acid phenethyl ester (CAPE), an active compound from New Zealand honeybee propolis. Whereas Wi-A was cytotoxic to both cancer and normal cells, CAPE has been shown to cause selective death of cancer cells. In the present study, we investigated the efficacy of Wi-A, CAPE, and their combination to ovarian and cervical cancer cells. Both Wi-A and CAPE were seen to activate tumor suppressor protein p53 by downregulation of mortalin and abrogation of its interactions with p53. Downregulation of mortalin translated to compromised mitochondria integrity and function that affected poly ADP-ribose polymerase1 (PARP1); a key regulator of DNA repair and protein-target for Olaparib, drugs clinically used for treatment of breast, ovarian and cervical cancers)-mediated DNA repair yielding growth arrest or apoptosis. Furthermore, we also compared the docking capability of Wi-A and CAPE to PARP1 and found that both of these could bind to the catalytic domain of PARP1, similar to Olaparib. We provide experimental evidences that (i) Wi-A and CAPE cause inactivation of PARP1-mediated DNA repair leading to accumulation of DNA damage and activation of apoptosis signaling by multiple ways, and (ii) a combination of Wi-A and CAPE offers selective toxicity and better potency to cancer cells.

## 1. Introduction

Withaferin A (Wi-A) is a steroidal lactone found in *Withania somnifera* (Ashwagandha), a popular ayurvedic herb. It has been shown to be cytotoxic to a variety of tumor cells [1,2,3,4,5,6,7,8,9]. Multiple mechanisms of its action have been described that include (i) induction of oxidative stress, reactive oxygen species (ROS) generation and loss of mitochondrial membrane potential yielding caspases and poly ADP-ribose polymerase (PARP) cleavage mediated apoptosis; (ii) inhibition of NF-kappa B signaling; (iii) targeting of Vimentin intermediate filaments, an essential protein involved in adhesion, migration, survival, and epithelial-mesenchymal transition (EMT); (iv) inhibition of AKT signaling; (v) induction and accumulation of p53 and its downstream proteins involved in growth arrest/apoptosis; (vi) downregulation of human papilloma virus (HPV) E6 and E7 oncoproteins; and (vii) inhibition of telomere lengthening process in ALT cancer cells [1,2,3,4,5,6,7,8,9,10,11,12,13,14,15,16].

The honeybee hive product propolis and its active ingredient caffeic acid phenethyl ester (CAPE) have also been assigned several pharmaceutical properties, including anti-inflammatory, immunostimulatory, anti-bacterial, anti-viral, and anticancer properties [17,18,19,20]. Several studies have shown that CAPE preferentially kills malignantly transformed cells and is relatively non-toxic to normal cells [21,22,23]. Apoptosis and differentiation are the two common endpoints reported for CAPE-treated cancer cells [24,25,26,27,28,29]. Furthermore, it has also been proposed to possess potent chemopreventive activity [21,30,31]. Multiple mechanisms of its action demonstrated in several laboratory studies, so far, include (i) inhibition of NF-kappa B and nitric oxide synthase (iNOS) signaling [26,32,33]; (ii) restoration of gap junctions and downregulation of p21^ras^ [34,35]; (iii) induction of p53, Bax and Bak yielding apoptosis [25,36,37]; (iv) inhibition of p21-activated kinase (PAK1), essential for the growth of both neurofibromatosis type 1 (NF1) and type 2 (NF2) [38]; (v) downregulation of mdr-1 responsible for drug resistance in cancer cells [39]; (vi) inhibition of Vascular endothelial growth factor (VEGF), a key regulator of angiogenesis, invasion and metastasis of cancer cells [40,41]; (vii) downregulation of Vimentin and Twist 2 that control EMT [42]; (viii) downregulation of Akt signaling, essential for cancer cell survival [43,44,45]; (ix) inhibition of histone deacetylase [46]; and (x) disruption of mortalin-p53 complexes leading to nuclear translocation and activation of p53 resulting in growth arrest in cancer cells [20]. Several studies have shown that CAPE causes decrease in cell migration, mediated by downregulation of tissue inhibitor of metalloproteinases-2 (TIMP-2), matrix metalloproteinases-2 (MMP-2), MMP-9, and mortalin [20,47,48,49]. It has also been shown to sensitize cancer cells to IR and other anticancer drugs [50,51] as well as protect normal tissues against their adverse effects. CAPE was shown to act both as radioprotector and radiosensitizer [52]. Lee et al. reported that pre-treatment with CAPE prior to the administration of t-BHP prevented hepatotoxicity [53]. Albukhari et al. showed protective effects of CAPE against Tamoxifen (TAM)-induced hepatotoxicity [54]. Motawi et al. also reported that it improves anticancer activity of TAM [55,56]. On the other hand, it attenuated the inhibition of neuritogenesis and downregulation of markers of neuroplasticity induced by cisplatin treatment [29]. Similarly, Matsunaga et al. reported the effectiveness of CAPE on cytotoxicity of doxorubicin and cisplatin; commonly used anticancer drugs. CAPE caused sensitization of cancer cells to these drugs and was suggested to be a potent adjuvant [57].

Ovarian and cervical cancers, the most common cancers among women worldwide, show high incidence of recurrence and are the top cause of death among gynecological malignancies. The treatments, including surgery, radiotherapy, and chemotherapy, are expensive and often complicated by several adverse side-effects and drug resistance. Poly ADP-ribose polymerase (key component of DNA repair processes) inhibitors (PARPi) (Olaparib, Rucaparib, and Niraparib) are the approved drugs for these cancers. Although the oral formulation of these inhibitors is attractive to patients, their adverse effects such as nausea and fatigue that impact quality of life [58] and high cost (~ $14,000 USD/month) [59] are of high concern. Natural products, on the other hand, are easily available, affordable, and considered less toxic alternative and/or combinational therapeutic modules. With these in mind, we performed bioinformatics and experimental analyses on the molecular effect of Wi-A and CAPE, and formulated their low dose combination. We demonstrate that Wi-A and CAPE, (i) in addition to the activation of tumor suppressor protein p53, mimic the activity of PARP1 inhibitor, Olaparib, and (ii) their low dose combination provides higher efficacy in these mechanisms.

## 2. Results

### 2.1. Wi-A and CAPE Caused Cytotoxicity to Cervical and Ovarian Cancer Cells

Several earlier studies have reported that the cytotoxicity of Wi-A and CAPE to cancer cells is mediated, at least in part, by targeting mortalin-p53 interactions [7,9,10,20] and reactivation of wild type p53 activities. We used abrogation of mortalin-p53 interaction and reactivation of p53 as an assay to screen for anticancer drugs from a library of small molecules including natural and synthetic compounds. The selected compounds were tested for their cytotoxicity to a variety of human cancer cells. We found that breast, cervical and ovarian cancer cells showed higher cytotoxicity as compared to the others including lung, prostate, bone and pancreatic cancer [60]. In this regard, we selected four cervical (SKGII, SKGIIIb, ME180 and HeLa) and two ovarian (SKOV3 and OKV-18) cancer cell lines and investigated their response to Wi-A and CAPE.

**Figure 1:**
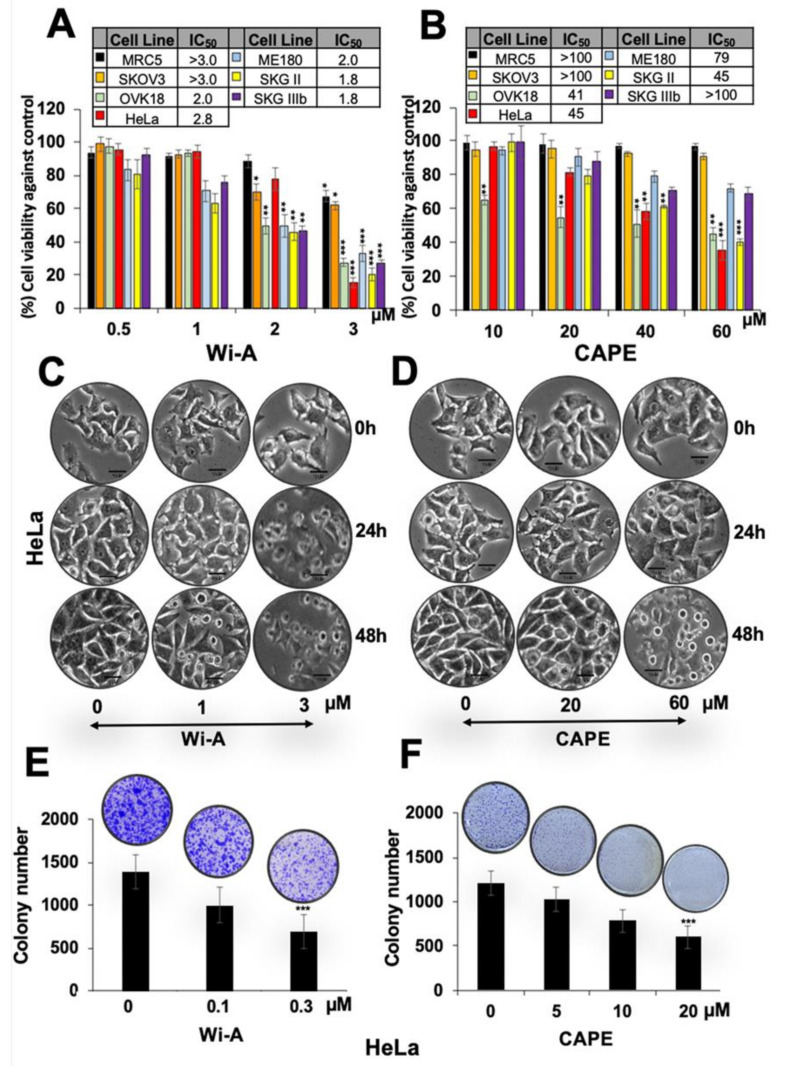
Dose dependent cytotoxicity of Withaferin A (Wi-A) and caffeic acid phenethyl ester (CAPE) to cervical and ovarian cancer cells. (**A**,**B**) 3-(4,5-dimethylthiazol-2-yl)-2,5-diphenyl tetrazolium bromide (MTT)-based cell viability assay showing cytotoxicity in various cell lines treated with Wi-A (**A**) or CAPE (**B**). IC_50_ values for each of the cell types used are shown in Table inset in A and B. (**C**,**D**) Phase contrast images showing cell morphology at 24 h and 48 h treatment showing predominant growth arrest and apoptosis, respectively, when treated with Wi-A (**C**) or CAPE (**D**); Scale bar-10 μM. (**E**,**F**) Long-term viability assay showing dose-dependent decrease in number of colonies (clonogenicity) for cells treated with either Wi-A (**E**) or CAPE (**F**). Data were normalized against the control and plotted as percent difference. Each data set represents the mean ± SD for at least three independent biological replicates. Statistical significance was defined as *p*-values (*) where * < 0.05, ** < 0.01 and *** < 0.001 represent significant, very significant and highly significant, respectively.

Using serial doses of Wi-A and CAPE, we found that Wi-A was cytotoxic to all these cell lines with half maximal inhibitory concentration (IC_50_) of 2–3 µM (Figure 1A). CAPE, on the other hand, showed less toxicity (IC_50_ was 45 µM for HeLa and SKGII, 41 µM for OKV-18, 79 µM for ME180, ~100 µM for SKOV3 and SKGIIIb) (Figure 1B). Mean IC_50_ values, calculated by plotting cell survival (%) versus drug concentration (µM), from more than three independent experiments are shown in Figure 1A,B. Normal human cells (MRC5) did not show toxicity to Wi-A at doses <2 µM and CAPE at doses <60 µM. Interestingly, among the different cell lines examined, SKOV3 showed weak response. On the other hand, HeLa, OVK18, and SKGII showed strong cytotoxicity in several independent experiments (Figure 1A,B). Furthermore, CAPE showed selective cytotoxicity to cancer cells (Figure 1B). Based on these data, we selected HeLa cells for further analysis. Microscopic observations of control and treated cells showed growth arrest at low dose and apoptosis at high dose (Figure 1C,D). Long-term viability assays showed dose-dependent decrease in colony-forming efficacy of cells treated with Wi-A or CAPE. Based on these short- and long-term viability analyses, we selected HeLa for further investigations on the molecular mechanism(s) of Wi-A and CAPE toxicity.

### 2.2. Wi-A and CAPE Caused Downregulation of Mortalin and Activation of Tumor Suppressor p53 Protein 

**Figure 2:**
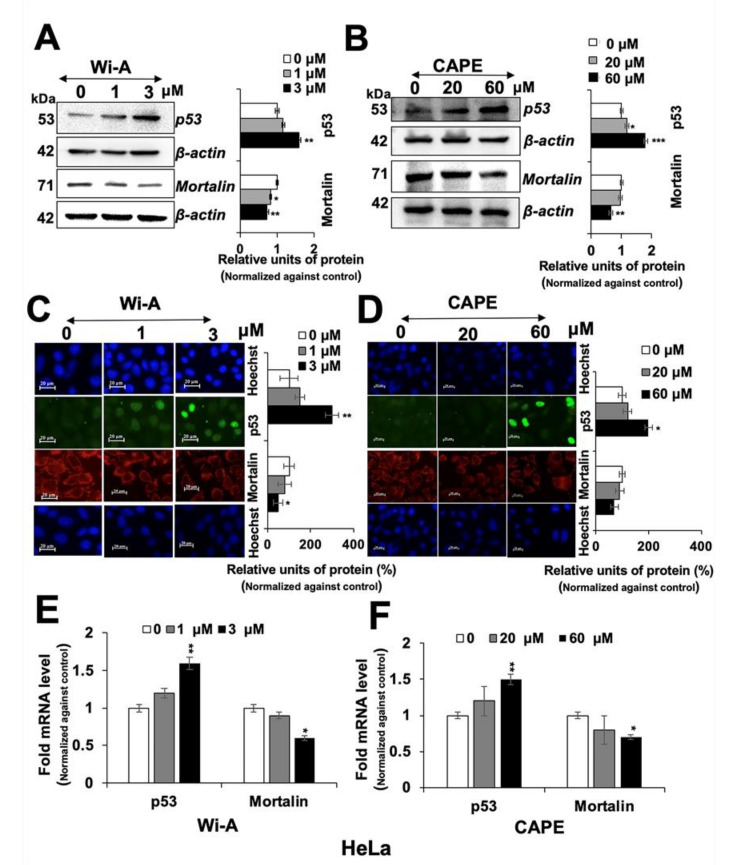
Withaferin A (Wi-A) and caffeic acid phenethyl ester (CAPE) caused downregulation of mortalin and activation of tumor suppressor p53 protein. Wi-A- and CAPE-treated cells showing dose-dependent decrease in mortalin and increase in p53 protein levels in HeLa cells as detected by Western blotting (**A**,**B**) and immunostaining (**C**,**D**); Scale bar–20 μM. Dose-dependent increase in p53 mRNA and decrease in mortalin mRNA were detected in cells treated with Wi-A (**E**) and CAPE (**F**). Data were normalized against the control and plotted as fold or percent difference as indicated. Each data set represents the mean ± SD for at least three independent biological replicates. Statistical significance was defined as *p*-values (*) where * < 0.05, ** < 0.01 and *** < 0.001 represent significant, very significant and highly significant, respectively.

Wi-A and CAPE have earlier been shown to target mortalin-p53 interactions in U2OS cells causing nuclear translocation and activation of tumor suppressor p53 protein [61]. We examined control, Wi-A and CAPE treated HeLa cells for p53 and mortalin expression. As shown in Figure 2A,B, p53 protein levels showed clear increase in the Western blots. Immunostaining of control and treated cells with specific antibodies for p53 and mortalin revealed increase in nuclear p53 (Figure 2C,D). Analysis of p53 and mortalin mRNA revealed dose dependent increase in p53 and decrease in mortalin mRNA (Figure 2E,F) in treated cells as compared to the untreated counterparts, suggesting that these compounds not only affect mortalin-p53 interaction at protein level but also regulate their level of expression at the transcriptional level that may translate to their multiple modes of action.

### 2.3. Wi-A and CAPE Triggered PARP1 Cleavage and Apoptosis Signaling

We next examined the control and treated cells for proteins involved in DNA repair and apoptosis signaling.

**Figure 3:**
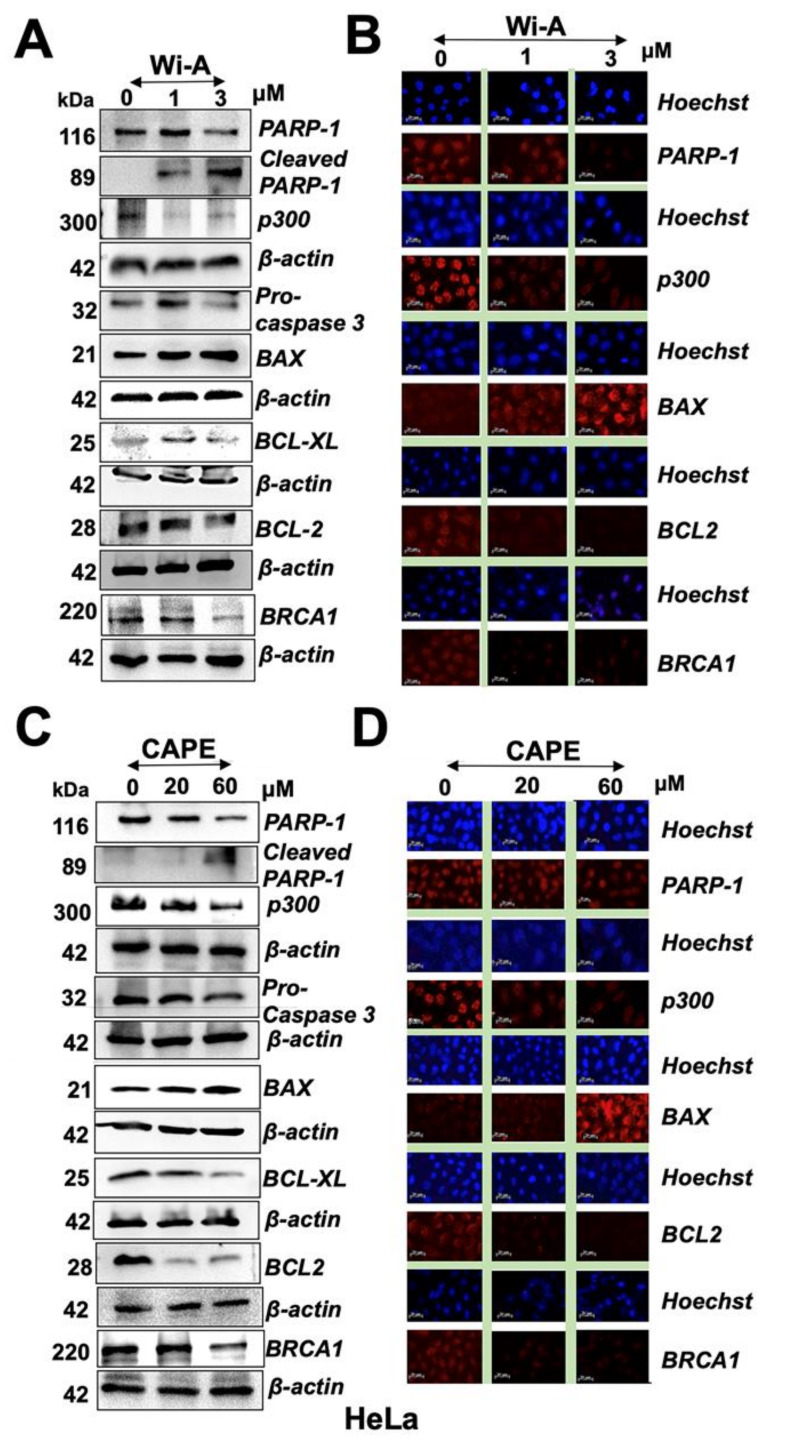
Withaferin A (Wi-A) and caffeic acid phenethyl ester (CAPE) triggered poly ADP-ribose polymerase1 (PARP1) cleavage and apoptosis signaling. Wi-A (**A**,**B**) and CAPE (**C**,**D**)-treated cells showed decrease in PARP1, p300, pro-caspase 3, B-cell lymphoma 2 (Bcl-2), B-cell lymphoma-extra large (Bcl-xL), and breast cancer type 1 susceptibility protein (BRCA1) as detected by Western blotting (**A**,**C**) and immunostaining (**B**,**D**); Scale bar-20 μM. Each data set represents the mean ± SD for at least three independent biological replicates.

As shown in Figure 3A,C and Figure S1, both Wi-A and CAPE caused induction of PARP1 cleavage that was clearly associated with activation of apoptosis signaling including cleavage of procaspase, increase in Bcl-2-associated X protein (Bax), and decrease in B-cell lymphoma 2 (Bcl-2) and B-cell lymphoma-extra large (Bcl-xL). The results were supported by immunostaining with specific antibodies (Figure 3B,D and Figure S1). Furthermore, decrease in BRCA1 was detected both in Wi-A and CAPE treated cells as compared to their untreated counterparts, and was consistent with earlier reports [62,63]. We next investigated if reduction in mortalin and PARP1 in Wi-A/CAPE-treated cells was interlinked. Cells were compromised for mortalin by mortalin-specific shRNA. As expected, mortalin-compromised cells showed increase in p53. Of note, these showed induction of PARP1 cleavage and a decrease in p300 and BRCA1 (Figure S2A). We also examined the effect of Olaparib and C464 (specific inhibitor of PARP1/2 and p300, respectively) and found that both cause a decrease in mortalin and an increase in p53 (Figure S2B,C). These data showed that PARP1 and mortalin-p53 pathways are closely related.

### 2.4. Combination of Wi-A and CAPE Possesses Stronger Cytotoxicity

**Figure 4:**
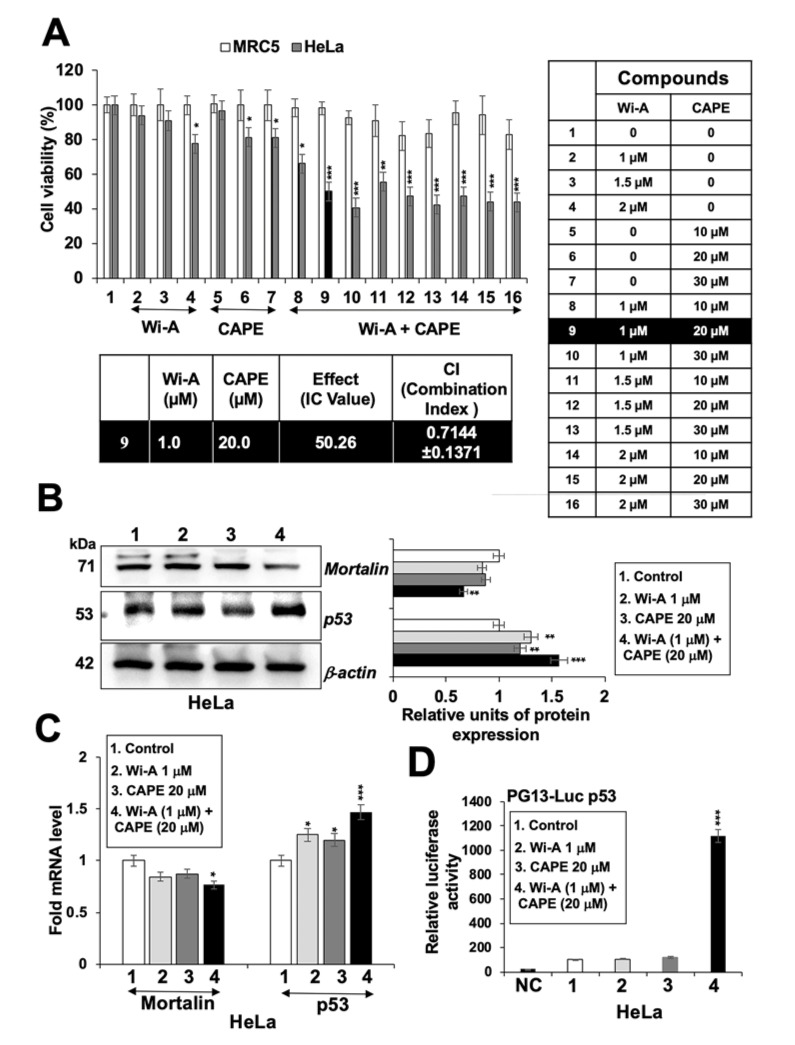
Combination of Withaferin A (Wi-A) and caffeic acid phenethyl ester (CAPE) possesses stronger cytotoxicity. 3-(4,5-dimethylthiazol-2-yl)-2,5-diphenyl tetrazolium bromide (MTT)-based cell viability assay showing dose dependent cytotoxicity either Wi-A or CAPE alone, or in combination in MRC5 and HeLa cells (**A**). Wi-A, CAPE, and their combination showed decrease in mortalin and increase in p53 protein (**B**) and mRNA levels (**C**). Wild type p53 protein-driven luciferase reporter (PG13-Luc) assay showed remarkable increase in cells when treated with the combination (**D**). Data were normalized against the control and plotted as fold and percent difference. Each data set represents the mean ± SD for at least three independent experiments. Statistical significance was defined as *p*-values (*) where * < 0.05, ** < 0.01 and *** < 0.001 represent significant, very significant and highly significant, respectively.

**Figure 5:**
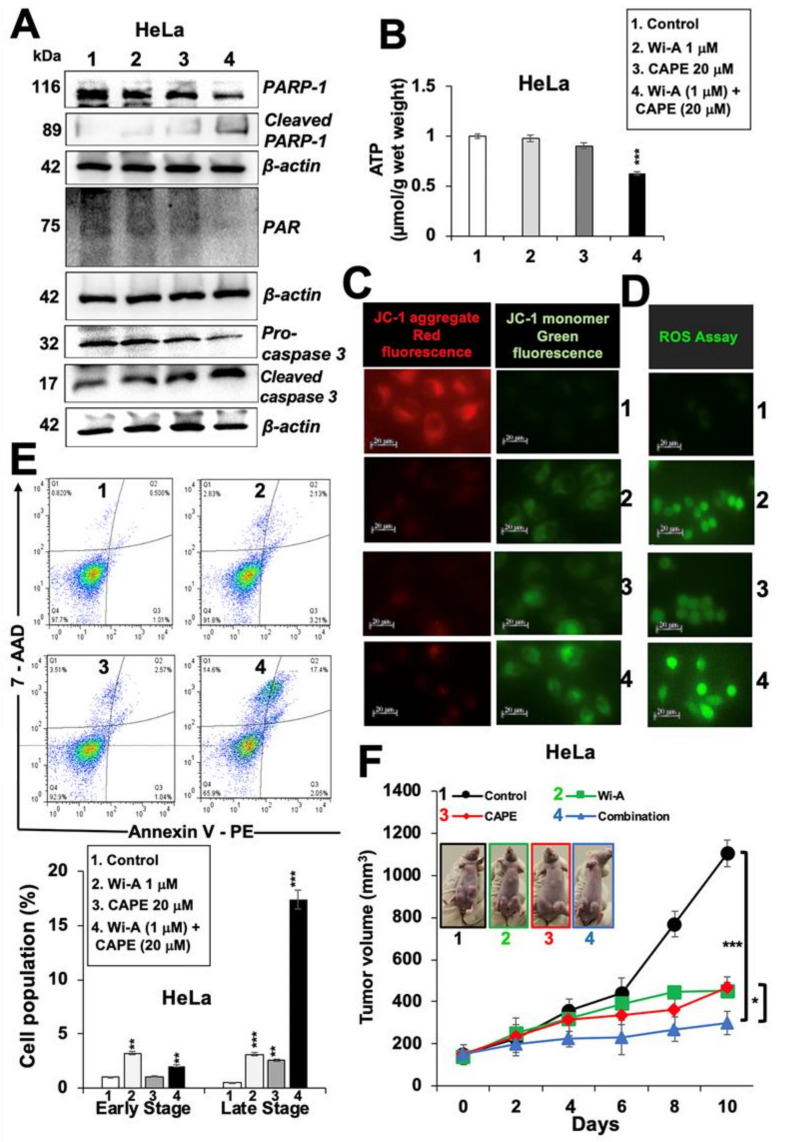
Withaferin A (Wi-A) and caffeic acid phenethyl ester (CAPE) in combination caused stronger activation of apoptosis signaling. (**A**) Western blot analysis of Wi-A, CAPE, and their combination treated cells showing stronger decrease in poly ADP-ribose polymerase1 (PARP1), poly (ADP-ribose) polymerase (PAR), and Pro-caspase 3 levels in the latter (**A**). Whereas Wi-A- and CAPE-treated cells did not show decrease in adinosine triphosphate (ATP) levels, the combination showed decrease (**B**). Wi-A, CAPE, and their combination treatment showed lower mitochondrial membrane potential as detected by JC-1 staining (**C**); Scale bar–20 μM and increase in oxidative stress as detected by reactive oxygen species (ROS) (**D**); Scale bar–20 μM. Flow cytometric analysis showing increase in apoptotic cells when treated with the combination as compared to either Wi-A or CAPE alone (**E**); quantification of E is shown below. Effect of Wi-A, CAPE, and their combination on tumor growth of subcutaneous xenografts revealed strong suppression achieved by the later (**F**). Each data set represents the mean ± SD for at least three independent biological replicates. Statistical significance was defined as *p*-values (*) where * < 0.05, ** < 0.01 and *** < 0.001 represent significant, very significant and highly significant, respectively.

As shown above, Wi-A showed stronger cytotoxicity and efficacy (IC_50_: 1–3 µM) than CAPE (IC_50_: 40–100 µM) to cancer cells. Based on these, we hypothesized that it may be possible to generate a combination of Wi-A and CAPE that might be more potent and selective to cancer cells. Based on the dose dependent and titration of viability by three independent viability assays (3-(4,5-dimethylthiazol-2-yl)-2,5-diphenyl tetrazolium bromide (MTT), Water soluble tetrazolium salt (WST), and Crystal violet (CV)) (Figure 4A, Figure S3A), we selected combination of Wi-A (1 µM) and CAPE (20 µM) and observed cytotoxicity in HeLa, ME180, SKGII, SKGIIIb, and OVK18 cells, whereas normal cells were safe at this combination. We next explored whether the combination of Wi-A and CAPE could synergistically enhance cytotoxic effect. Combination Index (CI) analysis revealed that the combination of Wi-A and CAPE exerted synergistic anti-proliferative effect, as evidenced by CI value 0.7144 (less than 1) (Figure 4A). To exclude selectivity criteria toward a particular cancer cell line, we used other ovarian and cervical cancer cell lines and performed cell viability assay (Figure S3B) and found stronger cytotoxic effect in the combination of Wi-A and CAPE as compared to individual compound in all the cell lines examined. We next examined the effect of combination on mortalin-p53 interaction and PARP1 signaling. As shown in Figure 4B,C, Wi-A + CAPE caused a stronger decrease in mortalin and an increase in p53 at both protein and transcript levels, respectively. Consistent to these results, in wild type p53 reporter assays, Wi-A + CAPE treated cells showed several folds higher p53 activity as compared to the cells treated with either Wi-A or CAPE alone (Figure 4D). The data supports the synergistic effect of the combination as suggested by the CI analysis. Furthermore, we examined the cells for DNA repair and apoptosis markers. As shown in Figure 5A, Figure S4A, Wi-A + CAPE treatment resulted in higher level of PARP1 cleavage, decrease in poly (ADP-ribose) polymerase (PAR) and increase in cleaved Caspase as compared to the ones treated with either Wi-A or CAPE. The results were confirmed by immunostaining.

In order to elucidate the mechanism of action further, we hypothesized that a decrease in mortalin may lead to compromised mitochondrial function and oxidative stress to the treated cells. To test this, we investigated the level of adenosine triphosphate (ATP) in control and treated cells. As shown in Figure 5B, Wi-A + CAPE-treated cells showed a decrease in ATP; the cells treated with a low dose of either Wi-A or CAPE alone did not show such an ATP decrease. Furthermore, assay for mitochondrial membrane potential (MMP) revealed decreased MMP in treated cells (Figure 5C). At the same time, the level of ROS was higher in the latter (Figure 5D), which reasoned for higher number of apoptotic cells (Figure 5E). In order to evaluate the tumor suppression efficacy of the combination with respect to each of the individual component, we recruited subcutaneous xenografts in nude mice. HeLa cells, initially tried, did not make tumors in nude mice. We then used SKOV3 cells. Subcutaneous SKOV3 xenografts generated tumors buds five days post-injection of cells. As shown in Figure 5F, control mice showed rapid growth of tumors during next 10 days; Wi-A and CAPE treated mice showed clear suppression of tumor growth early on. Of note, combination resulted in stronger tumor suppression and was in line with the in vitro data. No change in either the body weight or any other adverse effect was observed for all the groups during the course of the experiment.

**Figure 6:**
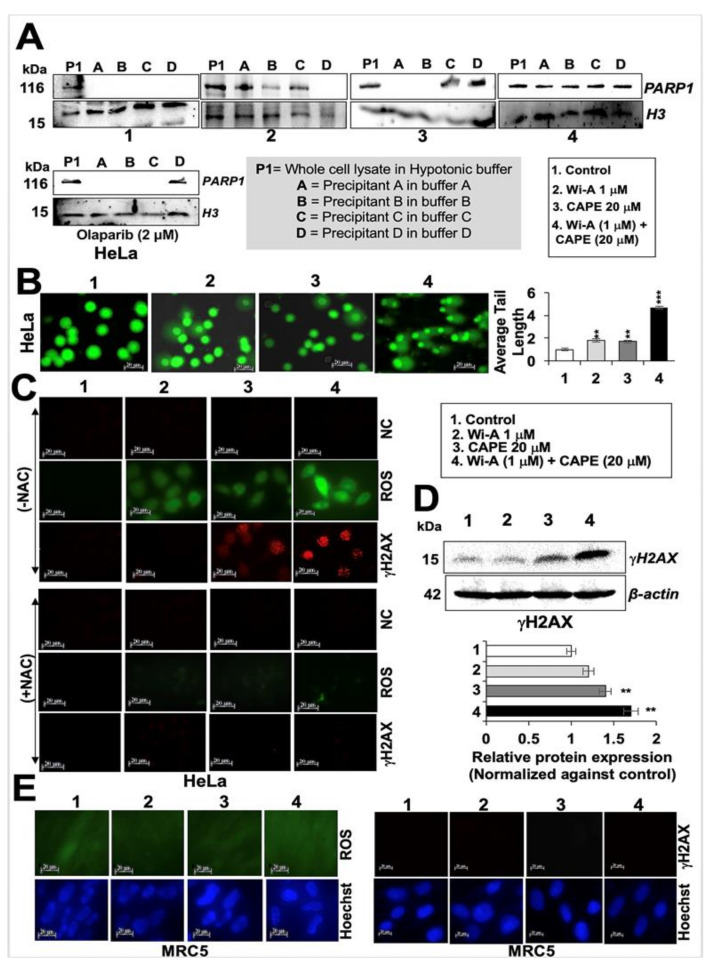
Withaferin A (Wi-A) and caffeic acid phenethyl ester (CAPE) directly interact with PARP1 and inhibit its function by DNA trapping. Western blot analysis of PARP1-DNA complexes showing trapping of PARP1 in DNA in Wi-A, CAPE, and their combination treated cells (**A**). Stronger trapping was observed in cells treated with the combination; Olaparib was used as a positive control. Alkaline comet assay showing accumulation of DNA damage in cells treated with Wi-A, CAPE and their combination (**B**); Scale bar–20 μM; the latter showed a remarkable increase. Immunostaining of H2A histone family member X (γH2AX) in control or in treated cells with either Wi-A or CAPE or their combination showing sharp increase in the number of γH2AX foci in the latter (**C**- *above*); cells pretreated with NAC showed abrogation in induction of ROS and γH2AX (**C**- *below*). Negative control (NC- *above*) shows staining with first antibody only; Scale bar–20 μM. Western blot showing increase in γH2AX in Wi-A, CAPE, and their combination treated cells (**D**). Immunostaining of normal cells treated with Wi-A, CAPE, and their combination is shown (**E**); Scale bar–20 μM. Data were normalized against the control and plotted as fold difference. Each data set represents the mean ± SD for at least three independent biological replicates. Statistical significance was defined as *p*-values where ** < 0.01 and *** < 0.001 represent very significant and highly significant, respectively.

In order to investigate the mechanism of action further, we next investigated whether decrease in mitochondrial function compromise PARP1 function, and thus performed a PARP1-DNA trapping assay. As shown in Figure 6A, PARP1 was trapped into the DNA in treated cells and was remarkably stronger in cells treated with Wi-A + CAPE as compared to the ones treated with either Wi-A or CAPE or Olaparib (a known PARP1 inhibitor). Inhibition of DNA repair in treated cells was endorsed by (i) Comet assay that showed accumulation of DNA double strand breaks (Figure 6B) and (ii) H2A histone family member X (γH2AX) that showed the DNA damage foci and significant increase in immunostaining and Western blotting, respectively in cells treated with combination of Wi-A and CAPE (Figure 6C,D). We next used N-acetyl cysteine (NAC), a known ROS inhibitor, to examine if the induction of DNA damage and production of ROS was linked in treated cells. As shown in Figure 6C and Figure S4B, whereas Wi-A+CAPE treated cells showed increase in both ROS and γH2AX (Figure 6C,D), pre-treatment with NAC inhibited their increase. On the other hand, equivalent treatment in normal (MRC5) cells did not cause induction of either ROS or γH2AX (Figure 6E). These data endorsed that the selective toxicity of Wi-A + CAPE combination to cancer cells is mediated, at least in part, through ROS and DNA damage pathways.

### 2.5. Wi-A and CAPE Directly Interact with PARP1

**Figure 7:**
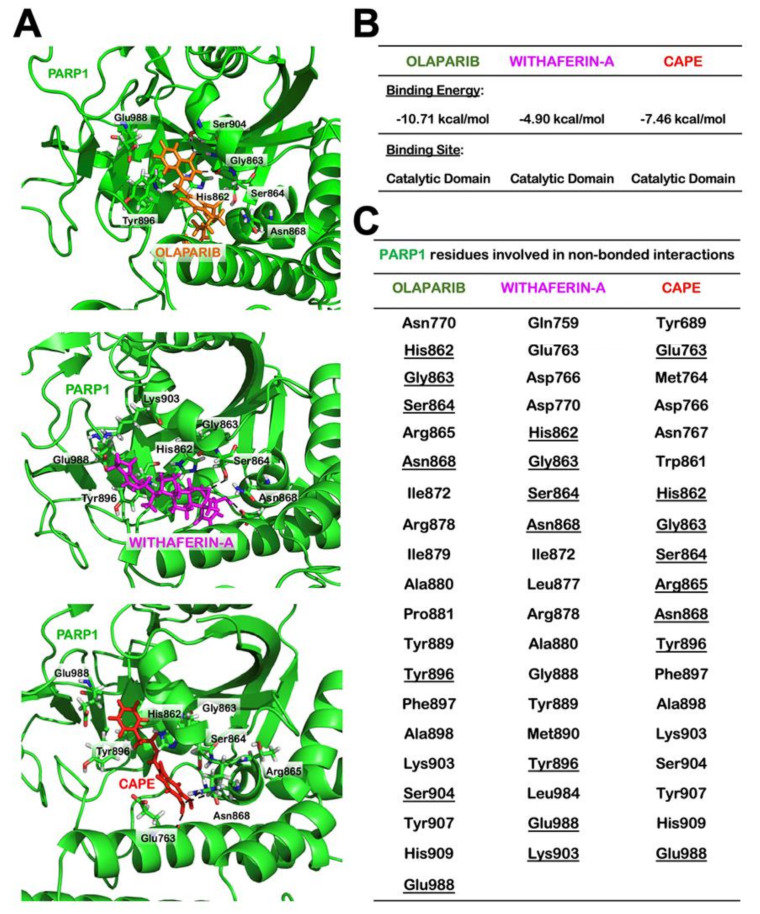
Molecular docking showing interactions of Withaferin A (Wi-A), caffeic acid phenethyl ester (CAPE), and Olaparib with poly ADP-ribose polymerase1 (PARP1). Wi-A and CAPE docked to PARP1 at the cavity lined by the catalytically active residues of PARP1, which was similar to the binding of Olaparib to PARP1 (**A**). Calculated binding energies and (**B**) details of PARP1 residues involved in non-bonded interactions (**C**).

As Olaparib directly interacts at the catalytic site of PARP1, we next used computational approach to find whether or not Wi-A and CAPE possessed the similar potential. For using Olaparib as a positive control, we first estimated a score for the binding affinity of Olaparib for PARP1. The docking score obtained was –10.71 kcal/mol. The molecular interactions in the docked complex (Figure 7) were quite similar to the interactions observed in PARP1-Olaparib PDB structure (PDB ID: 5DS3). It was in line with another computational study, where Autodock was used to study the interaction of Olaparib with PARP1 and the docking score obtained was −12.5 kcal/mol [64]. In both cases, the residues of PARP1 involved in polar interactions with Olaparib were Gly863 and Ser904. Therefore, we next docked Wi-A and CAPE in the same cavity and studied the interactions involved in the complex formation. The docking score for PARP1-Wi-A and PARP1-CAPE was found to be −4.90 kcal/mol and −7.46 kcal/mol, respectively. Though binding affinity of Wi-A was little less as compared to CAPE, the molecular interaction pattern for both the ligands was quite similar to that observed in PARP1-Olaparib complex (Figure 7). This data suggested that Wi-A and CAPE do possess the capability to interact directly with PARP1 at its catalytic site.

## 3. Discussion

Anticancer activity of *Withania somnifera,* has been assigned to Wi-A and Wi-N by a large number of independent studies that revealed their multiple targets and modes of action [4,7,9,65,66,67,68]. Furthermore, whereas Wi-A, by itself, caused toxicity to normal cells [7]; acute toxicity and pharmacokinetics studies revealed that in vivo oral administration of Wi-A in Ehrlich ascites carcinoma swiss mice model produces severe toxicity effects like diarrhea, weight loss and mortality above 70 mg/kg BW [69]. In humans, patients with high grade osteosarcoma showed good safety profile with oral Wi-A administration. Although the maximum tolerated dose was not achieved but 216 mg/day appeared as well tolerated dose by the patients with few side effects like skin rashes, fever, fatigue, edema, diarrhea and elevated liver enzymes [70]. Wi-A and its combination with Wi-N [7,71] and cisplatin [72] were shown to have better effects including stronger tumor suppressor, anti-metastasis, lesser drug resistant and tumor relapse, respectively. On similar lines, combination of Wi-N and Cucurbitacin B was shown to evoke senescence, selectively, in cancer cells [73]. These studies have endorsed that the combinational approach may provide a safer and effective alternative for cancer treatment. CAPE, an anticancer ingredient from honeybee propolis, has been shown to cause selective killing of cancer cells and inhibit in vivo tumor growth [22,23,74] through multiple modes of action [20,23,26,46,47,49,75,76]. In view of the low efficacy of CAPE, its several derivatives have also been suggested that may provide new and better therapeutic anticancer reagents [25,77]. These were also suggested to be effective for patients with varying p53 mutations in a more specific, effective, and targeted fashion. Interestingly, propolis, when normalized for CAPE content was shown to be more potent than CAPE alone [46]. CAPE also possessed the ability to enhance the anti-proliferative and cytotoxic effects of docetaxel and paclitaxel in prostate cancer cells [78] and tamoxifen in breast cancer cells [56] suggesting its potential for combinatorial therapy. In light of these data, whereas Wi-A and CAPE are limited by toxicity to normal cells and low efficacy, respectively [7,20,74,79], the combination of the two (safer for normal cells and high efficacy for cancer cells) was deemed useful for cancer therapeutic regimes.

As stated above, we generated a combination of Wi-A and CAPE in low doses and investigated its effect on cell phenotype and molecular signaling. Both of these have been shown to target the interaction of mortalin and p53 in cancer cells causing reactivation of tumor suppressor activity of p53 protein [7,8,9,10,20,80,81,82]. Whereas in all cancer cells (harboring wild type p53) the abrogation of mortalin-p53 interaction caused growth arrest, the cells with mutant p53 showed apoptosis [7,8,9,10,20,80,81,82]. Furthermore, although cells harboring different p53 mutants responded to Wi-A, cells with p53^Y220C^ mutation showed strong response that was attributed to the reversion of Y220C structural distortions and gain of wild type like tumor suppressor activity [9]. HeLa cells possess HPV virus whose E6 protein binds and degrades p53 through ubiquitin pathway [83]. Wi-A has been shown to downregulate expression of HPV-E6 protein and restore p53 in HeLa cells, resulting in apoptosis [16]. Furthermore, besides p53, Wi-A has been shown to activate ROS, c-Jun N-terminal kinase (JNK) and stabilize TP73 by phosphorylation in p53 compromised cells [84]. Taking our data together with existing literature [1,2,3,4,5,6,7,8,9,10,11,12,13,14,15,16,17,18,19,20], Wi-A and CAPE induced apoptosis is likely to be regulated through multiple signaling pathways. In the cytotoxicity assays, low dose combination showed selective toxicity to cancer as compared to normal MRC5 cells (Figure 4A and Figure S3A,B). The effect of low dose combination appeared to be synergistic as seen by calculating combination index. At the molecular level, we found it to target mortalin-p53 interaction and upregulate p53 tumor suppressor protein leading to growth arrest/apoptosis in cancer cells. Of note, the p53 activity and the growth arrest was stronger as compared to each of the reagent alone (Figure 4). Furthermore, we found that Wi-A, CAPE, and their combination caused decrease in mortalin and increase in p53 mRNA that may translate to activation of growth arrest and apoptosis signaling mediated by their multiple downstream effector proteins. Of note, consistent to the enhanced in vitro cytotoxicity of the combination (as compared to the individual components), in vivo tumor growth assays also revealed its stronger tumor suppressor activity (Figure 5).

We next examined the molecular interactions of each of the compound with target molecules, as summarized in Figure S5A. It was found that Wi-A could stably interact with the mortalin-binding region of p53 (docking score: −2.008 kcal/mol); the residues involved in the formation of hydrogen bonds were found to be Gln 331 and Thr 329 (Figure S5B). However, CAPE showed interaction with the ATPase domain of mortalin (docking score: −2.396 kcal/mol). It stably interacted at the p53-binding region of mortalin. In average structure, it showed hydrogen bond formation with Ala 195 and pi-pi stacking with Tyr196 (Figure S5C). It is thus suggested that the interaction of Wi-A and CAPE with the critical residues forming the mortalin-p53 interaction interface might be hindering the complex formation, therefore setting p53 free to translocate to nucleus and carry out its tumor suppressive transcriptional activation function. Further, Wi-A and CAPE might also inhibit the chaperonin activity of mortalin, as both the ligands showed stable interaction in the chaperone’s substrate binding cavity (Figure S5D,E, docking score: −1.975 kcal/mol and −6.949 kcal/mol). Additionally, Wi-A showed stable interaction in the mortalin-specific latch region (residues that act as a latch between lid and cleft region of the protein) as well (Figure S5F, docking score: −3.036 kcal/mol). These data suggest that Wi-A and CAPE would not compete for binding to residues forming the mortalin-p53 interaction interface. Different interacting partners and modes of binding, as far as abrogation of mortalin-p53 complex formation is concerned, may be attributed to their synergy and stronger activity as observed in our experiments.

Of note, the cells treated with combination, as compared to untreated control or treated with individual reagent, showed a stronger decrease in mortalin at both the protein and transcriptional levels. Such a decrease in mortalin may further push the reactivation of p53 in treated cells. Furthermore, since mortalin has multiple functions essential for cell survival, including maintenance of mitochondrial integrity, ATP production, and chaperoning [82,85], we hypothesized that such decrease in mortalin and associated mitochondrial dysfunction, increased oxidative stress may translate to apoptosis. Molecular analysis on apoptosis signaling revealed inhibition of PARP1 signaling, an essential component of DNA repair process. PARP1 is an established sensor of DNA single strand breaks and executes the process of DNA repair along with several other proteins (Figure 5 and Figure 6). Comet assay revealed negligible/ low level of increase in DNA fragmentation in cells treated with either Wi-A or CAPE, consistent with the earlier report [84]. Of note, the combination caused appreciable increase in DNA damage. PARP1, due to its amplification in a large variety of cancers and essential role in DNA damage repair, has emerged as a potent anticancer target [86,87,88,89]. Inhibition of PARP1 leads to accumulation of single and double strand DNA breaks resulting in genomic instability and apoptosis in cancer cells [90]. We next investigated whether downregulation of mortalin and inhibition of PARP1 were linked. As shown in Figure S2, mortalin-compromised cells afflicted PARP1 signaling and entered apoptosis. Cells treated with either Wi-A or CAPE and more specifically with the combination showed cleavage of PARP1, its trapping into the DNA and accumulation of double strand DNA breaks (Figure 5 and Figure 6). ROS are a group of short-lived, highly reactive, and oxygen-containing radicals that can induce DNA damage signaling. In our previous study, we had demonstrated that Wi-A treatment caused ROS activation and induction of γH2AX [7]. Whereas increase in γH2AX protein expression was seen in MCF7 cells treated with 1 µM Wi-A, also reported earlier [7,91], HeLa cells showed milder response (IC_50_ >2 µM) (Figure 1A). Wi-A at a 2 µM dose caused only ~20% reduction in viability in HeLa cells (Figure 1A) demonstrating differential response of different cell lines to Wi-A. Nevertheless, pre-treatment of cells with ROS inhibitor (NAC) abrogated the effect of Wi-A, CAPE, and Wi-A + CAPE on activation of DNA damage response suggesting essential role of ROS in anticancer activity of these compounds. Taken together, these results showed that Wi-A caused oxidative stress leading to ROS generation. This was in line with a previous report that also showed that activation of, c-Jun N-terminal kinase/Activator protein 1 (JNK/AP-1) mediated apoptosis signaling in response to ROS [92]. Other studies have reported that Wi-A induces inhibition of proteasomal degradation resulting in accumulation of ER chaperones (BiP and GRP94) and cytoplasmic heat shock proteins [93]. Furthermore, Wi-A and mild heat shock treatment was shown to enhance the synergistic accumulation of HSP70 and HSP30 accumulation synergistically; similar effect was not observed for BiP and GRP94 [93]. Together with other reports [1,2,3,4,5,6,7,8,9,10,11,12,13,14,15,16], these data have endorsed that Wi-A has multiple targets and modes of action. In addition to the mechanisms described earlier, we report that Wi-A and CAPE are able to target PARP1 directly and in a manner similar to its known inhibitor, Olaparib (Figure 7). Taken together, we found that a low dose combination of Wi-A and CAPE-induced selective cytotoxicity in cancer cells by mechanisms mediated by downregulation of mortalin, activation of tumor suppressor p53 and inhibition of PARP1. The two compounds showed non-overlapping interactions with the target proteins, and their combination yielded stronger effect as compared to the each of them alone.

## 4. Materials and Methods

### 4.1. Cell Culture and Reagents

Human cervical cancer (HeLa, SKGII, SKGIIIb, and ME180), ovarian cancer (SKOV3 and OKV18), and normal lung fibroblast (MRC5) cells were purchased from the Japanese Collection of Research Bioresources (JCRB, Tokyo, Japan). All the cells lines were cultured in Dulbecco’s Modified Eagle’s Medium (DMEM) (Invitrogen, Carlsbad, CA, USA) supplemented with 5–10% fetal bovine serum (Fujifilm WAKO Pure Chemical Corporation, Osaka, Japan) in a humidified incubator (37 °C and 5% CO_2_). Wi-A and CAPE were dissolved in dimethyl sulfoxide (DMSO) (WAKO, Osaka, Japan) to make 5 mM stock and added to the complete cell culture medium, to obtain the working concentration as indicated. Cells grown at 60–70% confluency were treated with either Wi-A or CAPE or their combination for 48 h. For microscopic observations, cells were cultured in six-well plates, and we examined them live and in fixed states (as described below in immunocytochemistry section). Mortalin-targeting shRNA (shRNA-2166) was used for knockdown of mortalin as described previously [79,80]. It was transfected into cells using X-tremeGENE^TM^ 9 (Roche, Basel, Switzerland), following the manufacturers’ protocol. After 24–48 h, the transfected cells were subjected to Western blot analysis.

### 4.2. PG-13 Luciferase Reporter Assay

HeLa cells were seeded in 6-well plate and allowed to adhere overnight. Cells were transfected with wild-type p53 responsive luciferase reporter plasmid (PG13-Luc) (a kind gift from Professor Bert Vogelstein). Plasmid was transfected into cells using X-tremeGENE 9 (Roche, Basel, Switzerland). The transfected cells were treated with either Wi-A or CAPE or their combination for 48 h. The activity of luciferase was detected using Dual-Luciferase^®^ Reporter Assay System (Promega, Madison, WI, USA) following the manufacturer’s instructions. The luciferase activity was measured using Tecan infinite M200^®^ Pro microplate reader (Tecan Group Ltd., Mannedorf, Switzerland).

### 4.3. Cytotoxicity/Growth Inhibition Assay

Cytotoxicity of Wi-A, CAPE and their combination was evaluated by quantitative colorimetric assays using MTT (3-(4,5-dimethylthiazol-2-yl)-2, 5-diphenyltetrazolium bromide (Sigma Aldrich, Tokyo, Japan) assay, WST-1 Cell Proliferation Assay (TAKARA BIO, Shiga, Japan) and Crystal Violet (CV) staining. Cells (5 × 10^3^)/well were plated in 96-well plate and allowed to adhere overnight. Adhered cells were treated with Wi-A, CAPE and combination as indicated doses for 48 h. For quantitative colorimetric assay, MTT (0.5 mg/mL) solution was added to the cell culture medium for 4 h, after which the medium was replaced with DMSO (100 μL) for dissolution of formazan crystals. WST-1 (100 μL) solution was added to the cell culture medium for 4 h. For CV staining, cell medium was replaced with ice-cold methanol, incubated at 4 °C for 10 min and then replaced with CV stain followed by incubation at room temperature for 3 h. The stained cells were incubated with de-staining solution (15% methanol, 15% glacial acetic acid and MQ) until a homogenous color was obtained. Absorbance was recorded at 570 nm for MTT and CV assays, whereas for WST-1 absorbance was recorded at 450 nm, with 630 nm as reference using spectrophotometer (TECAN Group Ltd., Zurich, Switzerland). The standard deviation and statistical significance of the three independent experiments was determined by unpaired *t*-test using Graph Pad software (GraphPad Software, Inc, La Jolla, CA, USA).

### 4.4. Colony Formation Assay

Five hundred cells/well were plated in six-well plates and allowed to adhere overnight. After overnight incubation, cells were treated with Wi-A, CAPE and combination with indicated doses. The treated cells were then left to form colonies for the next 10–14 days. Control and drug-supplemented media was changed every three days. Colonies were monitored under the microscope, washed gently with PBS, fixed with pre-chilled acetone: methanol (1:1) at room temperature for 5–10 min, and then stained with 0.5% crystal violet solution (Sigma-Aldrich, C3886) at room temperature for 2 h. Dishes were washed with tap water to rinse off excessive crystal violet, dried, and photographed. Colonies were counted manually. Quantitation of the data was obtained from three independent experiments.

### 4.5. Western Blot Analysis

Control and treated cells were lysed using RIPA lysis buffer (Thermo Fisher Scientific, Rockford, IL, USA) containing complete protease inhibitor cocktail (Roche Applied Science, Mannheim, Germany) in cold room for 30 min. Lysates were centrifuged at 15,000 rpm for 15 min, and the supernatant was used for Western blotting with specific antibodies as indicated. The protein concentrations of cell lysate were measured by bicinchoninic acid assay (BCA) (Thermo Fisher Scientific, USA). The cell lysates containing 10–20 μg protein were separated on 8–12% SDS-polyacrylamide gel electrophoresis (SDS-PAGE) and transferred to a polyvinylidene difluoride (PVDF) membrane (Millipore, Billerica, MA, USA) using a semidry transfer blotter (ATTO Corporation, Tokyo, Japan). Membranes were blocked with 3% BSA (WAKO Japan) at room temperature for 2 h. Blocked membranes were probed with target protein-specific primary antibodies (1–3 μg/mL) including PARP1/2(H-250), p53(FL 393), p300(F-4), pro-caspase 3 (H-277), Bax (H20X), Bcl-2 (N-19), Bcl-xL (8362), Histone 3 (FL-136) (Santa Cruz Biotechnology, Paso Robles, CA, USA), BRCA1 (9010S), PARP1 (5625), PAR (ab14459), Caspase 3 (9661), γH2AX (9718S) (Cell Signaling Technology, Danvers, MA, USA), and β-actin (ab49900) (Abcam, Cambridge, UK) at 4°C overnight. Mortalin antibody was raised in our laboratory. The blots were incubated with the following secondary antibodies (0.5–1.0 μg/mL) conjugated to horseradish peroxidase: anti-rabbit IgG or anti-mouse IgG (Santa Cruz Biotechnology) and developed by enhanced chemiluminescence reaction (ECL) (GE Healthcare, Amersham, Buckinghamshire, UK). β-actin antibody (Abcam, Cambridge, UK) was used as an internal loading control. ImageJ (NIH, Bethesda, MD, USA) software was used to quantify protein signals.

### 4.6. Immunocytochemistry

Cells (1–3 × 10^3^/well) were plated on 18-mm glass coverslips placed in a 12-well culture plate. After 24 h incubation, cells were treated with Wi-A, CAPE, or their combination for 48 h and were then fixed in pre-chilled acetone: methanol (1:1) at 4 °C for 5–10 min. The fixed cells were washed thrice (10 min each time) with PBS, permeabilized by incubation with PBS-0.1% Triton X-100 for 10 min, blocked with 2% bovine serum albumin in PBST for 1 h, and then incubated with primary antibodies (1–3 μg/mL) overnight. The details of antibodies used are mentioned in the western blot analysis section. Immunostaining was visualized by secondary antibody staining. Secondary antibodies (1–2 μg/mL) conjugated with either Texas RED (Amersham Biosciences, Buckinghamshire, UK) or FITC, Alexa-488 or Alexa-594 (Molecular Probes, Eugene, OR, USA) were used as indicated. Hoechst (Invitrogen, Molecular Probes, Eugene, OR, USA) was used for nuclear staining. Cells were examined on a Zeiss Axiovert 200 M microscope by 40× objective lens and analyzed by AxioVision 4.6 software (Carl Zeiss, Tokyo, Japan). ImageJ (NIH, Bethesda, MD, USA) software was used to quantify fluorescence signals.

### 4.7. Comet Assay

DNA comet assay (Trevigen, Inc, Gaithersburg, MD, USA) was used to detect DNA strand breaks following the manufacturer’s instructions. Open Comet (v1.3) software (GNU General Public License) was used to calculate the percent DNA in tail. Image J was used for image processing.

### 4.8. ROS Assay

Cells were cultured on glass coverslips and stained for ROS detection using Image-IT^TM^ LIVE green ROS detection kit (Molecular Probes, Eugene, OR, USA following the manufacturer’s instructions. Images were captured using Zeiss Axiovert 200M microscope and analyzed by AxioVision 4.6 software (Carl Zeiss, Tokyo, Japan).

### 4.9. ATP Assay

ATP levels in control and treated cells were analyzed using Luminescent ATP detection assay kit (Abcam plc, Cambridge, UK; ab113849) following the manufacturer’s instructions.

### 4.10. Apoptosis Assay

Control and treated cells were collected by centrifugation at 3000 rpm at 4 °C for 5 mins. Floating cells were also pooled by centrifugation. The cell pellet was re-suspended with 100 μL fresh media and stained with Guava Nexin Reagent (EMD Millipore Corporation, Berlington, MA, USA). Apoptotic cells were quantified by FlowJo software (Version 7.6, Flow Jo, LLC, Ashland, OR, USA).

### 4.11. Trapping Assay

Control and treated cells were trypsinised with 0.5 mL of trypsin-EDTA and collected by centrifugation. Different stringency buffer (as follows) were used to perform PARP1 trapping. These included (i) Hypotonic buffer: 100 mM MES-NaOH pH 6.4, 1 mM EDTA, 0.5 mM MgCl_2_, 30% sucrose in MiliQ, (ii) Buffer A: 50 mM HEPES-NaOH pH 7.5, 100 mM KCL, 2.5 mM MgCl_2_, 0.05% Triton X-100, (iii) Buffer B: 50 mM HEPES-NaOH pH 7.5, 250 mM KCl, 2.5 mM MgCl_2_, 0.05% Triton X-100, (iv) Buffer C: 50 mM HEPES-NaOH pH 7.5, 500 mM KCL, 2.5 mM MgCl_2_, 0.1% Triton X-100, and (v) Buffer D: Buffer A, 5 mM CaCl_2_. Micrococcal protease inhibitor three-unit (Roche Diagnostic GmbH, Mannheim, Germany) was added to each of the five. Cell pellets were incubated with indicated buffer and vortexed for 10 min followed by centrifugation at 16,000 rpm at 4 °C for 10 min. The supernatant was collected and labelled as P1 and the pellet was re-suspended with buffer A followed by centrifugation at 16,000 rpm at 4 °C for 10 min. The step was repeated in the sequence of A–D buffers. Supernatant from each centrifugation was labelled as A, B, C, and D and were subjected to Western blotting using anti-PARP1/2 and anti-Histone H3 antibodies.

### 4.12. JC-1 Staining

Control and treated cells were incubated with JC-1 (Molecular probes) reagent for 30 min. Later, cells were washed with PBS and immediately processed for imaging using Zeiss Axiovert 200M microscope and analyzed by AxioVision 4.6 software (Carl Zeiss, Tokyo, Japan).

### 4.13. Combination Index (CI) Analysis

Drug dilutions and combinations were made in culture medium immediately before use. MTT, WST and CV staining assays were used to evaluate cell viability. Commercially available Chou and Talalay (Compusync software) was used to calculate CI [94]. Detailed instructions have been followed during CI calculation. According to the recommendation and methodology of this software, score of CI less than 1, greater than 1, or equal to 1 represent synergism, antagonism, or additive effect, respectively.

### 4.14. RNA Extraction and Quantitative Real-Time Polymerase Chain Reaction

Total RNA from control and treated cells was collected using RNeasy mini kit (Qiagen, Stanford Valencia, CA, USA). RNA (1 µg) was used for reverse transcription following the protocol from QuantiTect Rev, Transcription Kit (Qiagen, Tokyo, Japan). Quantitative real-time PCR was performed using Syber Select Master Mix (Applied Biosystem, Life Technologies, Foster City, CA, USA) method. The condition of qPCR was 50 °C for 2 min, 95 °C for 10 min, followed by 40 cycles of denaturing at 95 °C for 15 sec and annealing at 60 °C for 1 min. A melting curve was then generated to assess the specification of the PCR amplification. The geometric mean of housekeeping gene 18S was used as an internal control to normalize the variability in expression levels. Primers sequences are given in Table 1.

### 4.15. In Vivo Tumor Suppression

Effect of Wi-A, CAPE and their combination was examined by nude mice in vivo tumor growth assay using subcutaneous xenografts of SKOV-3 (of note, HeLa cells did not make tumors in nude mice). Female 4–5 weeks old BALB/c nude mice were bought from Nihon Clea (Shizuoka, Japan). All mice were fed on standard food pellet and water ad libitum, acclimatized to laboratory conditions (24  ±  2  °C), relative humidity of 55–65%, and 12 h light/dark cycle for 5–7 days. Cells, grown to 80–90% confluency were harvested by trypsinization and suspended in PBS. 5 × 10^6^ cells were injected subcutaneously (s. c.) on left and right flanks of mice (*n* = 4) in 100 µL PBS within 30 min of harvesting. Control and treatment groups were injected (intraperitoneally) with vehicle (phosphate buffered saline, PBS) or Wi-A (10 mg/kg body weight) or CAPE (100 mg/kg body weight) or combination (Wi-A + CAPE) every day for next 10 days. Mice were weighed, and tumor were measured using caliper every alternate day. Tumor volume was calculated using equation V = L × W^2^/2, where L is length and W is width. Average body weight and tumor volume from eight tumors/group was plotted. This study was carried out in strict accordance with the recommendations from the Animal Experiment Committee, Safety and Environment Management Division, National Institute of Advanced Industrial Science & Technology (AIST), Japan (Approval Number 2019-0025).

### 4.16. Bioinformatics Analysis

Docking of PARP1 with Wi-A, CAPE and Olaparib was performed using Glide from Schrodinger’s small-molecule drug discovery suite [95]. Molecular interaction of mortalin and p53 with Wi-A and CAPE was also studied. The structure of PARP1 (PDB ID: 5DS3), p53 (PDB ID: 1OLG) and mortalin (PDB ID: 4KBO and 3N8E) was obtained from RCSB PDB. As X-ray crystallographic structure of mortalin SBD (PDB: 3N8E) is not complete, homology modelling was used to model the C-terminal residues (598-648 aa), using the *E. coli* DNAK SBD (PDB: 1DKY) as a template. All the protein structures were prepared for docking experiment using protein preparation wizard of Schrodinger. The preparation steps mainly involved the addition and optimization of hydrogen atoms, filling of missing amino acid side chains, correction of bond orders and energy minimization. The structure of Wi-A (CID: 265237), CAPE (CID: 5281787), and Olaparib (CID: 23725625) was obtained from PubChem. These small molecules were further prepared using Ligprep that generates all possible confirmations of small molecules and minimizes their energy. For PARP1, the grid was generated at the catalytic site, following the mechanism of action of Olaparib [96]. In p53 structure, the small molecules were targeted at the mortalin-binding region (323-337). For interaction of Wi-A and CAPE with mortalin, both substrate binding and nucleotide binding domains were explored. The docked complexes were subjected to molecular dynamics (MD) simulations using Desmond as previously described [20] to investigate the stability of protein–ligand interactions. The molecular interaction pattern for each complex was studied by generating an average structure representing the stable trajectory of its MD simulation run.

### 4.17. Statistical Analysis

Data from three or more experiments were expressed as mean ± standard deviation. Unpaired *t*-test (GraphPad Prism GraphPad Software, San Diego, CA, USA) has been performed to determine the degree of significance between the control and experimental samples. Statistical significance was defined as significant (* *p*-value ≤ 0.05), very significant (** *p*-value ≤ 0.01) and highly significant (*** *p*-value ≤ 0.001).

## 5. Conclusions

In this study, we demonstrated that the anticancer activity of Wi-A and CAPE is mediated by downregulation of mortalin and PARP1, yielding upregulation of tumor suppressor p53 and DNA-damage signaling leading to growth arrest/apoptosis in cancer cells. We generated a low dose combination of Wi-A and CAPE that showed enhanced activity. Further studies on its efficacy to cancer cell metastasis, stemness, drug resistance in in vitro and in vivo models are warranted.

## Figures and Tables

**Table 1:** Sequences of primers used.

Gene (Human)	Sequence (5′-3′)
p53 forward	GTTCCGAGAGCTGAATGAGG
p53 reverse	TCTGAGTCAGGCCCTTCTGT
Mortalin forward	AGCTGGAATGGCCTTAGTCAT
Mortalin reverse	CAGGAGTTGGTAGTACCCAAATC
PARP1 forward	TCAGCCTCCTTGCTACAGAGG
PARP1 reverse	GGTCGTTCTGAGCCTTTAGGG
p300 forward	AAACCCACCAGATGAGGAC
p300 reverse	TATGCACTAGATGGCTCCGCAG
18S forward	CAGGGTTCGATTCCGTAGAG
18S reverse	CCTCCAGTGGATCCTCGTTA

## Supplementary Materials

The following are available online at https://www.mdpi.com/2072-6694/12/5/1160/s1, Figure S1: Quantitation of apoptosis markers modulated by Wi-A and CAPE treatment, Figure S2: Effect of mortalin on PARP1 and *vice versa*, Figure S3: WST and CV staining-based cell viability assay showing dose dependent cytotoxicity of either Wi-A or CAPE or their combination in HeLa cells. Cytotoxicity of Wi-A, CAPE and their combination to different ovarian and cervical cancer cells, Figure S4: Quantitation of apoptotic markers in control and treated (Wi-A, CAPE and combination) cells from data shown in Figure 5A. Quantitative measurement of fluorescence intensity of ROS and γH2AX in control and treated (Wi-A, CAPE and combination) cells, in presence or absence of NAC from data presented in Figure 6C, Figure S5: Molecular dockings and simulations to study the interaction of Wi-A and CAPE to target proteins, mortalin and p53, Figure S6. Western blot of protein of interest (p53 and mortalin) and respective. β-actin expression presented at Figure 2A, Figure S7. Western blot of protein of interest (p53 and mortalin) and respective β-actin expression presented at Figure 2B, Figure S8. Western blot of protein of interest (PARP1, Cleaved PARP1, p300, Pro-caspase 3 and Bax) and respective β-actin expression presented at Figure 3A, Figure S9. Western blot of protein of interest (Bcl-xL, Bcl-2 and BRCA1) and respective β-actin expression presented at Figure 3A, Figure S10. Western blot of protein of interest (PARP1, Cleaved PARP1, p300, and Pro-caspase 3) and respective β-actin expression presented at Figure 3C, Figure S11. Western blot of protein of interest (Bax, Bcl-xL and Bcl-2) and respective β-actin expression presented at Figure 3C, Figure S12. Western blot of protein of interest (BRCA1) and respective β-actin expression presented at Figure 3C, Figure S13. Western blot of protein of interest (Mortalin & p53) and respective β-actin expression presented at Figure 4B, Figure S14. Western blot of protein of interest (PARP1, Cleaved PARP1, PAR, Pro-caspase 3, and Cleaved caspase 3) and respective β-actin expression presented at Figure 5A, Figure S15. Western blot analysis of PARP1-DNA complex showing trapping of PARP1, and H3 as a control presented on Figure 6A, Figure S16. Western blot analysis of PARP1-DNA complex showing trapping of PARP1, and H3 as a control presented on Figure 6A, Figure S17. Western blot of protein interest (γH2AX) and respective β-actin expression presented on Figure 6D, Figure S18. Western blot of protein of interest (Mortalin) and respective β-actin expression presented on Figure 2A, Figure S19. Western blot of protein of interest (p300, PARP1, Cleaved PARP1, p53 and BRCA1) and respective β-actin expression presented on Figure S2A, Figure S20. Western blot of protein of interest (PARP ½, p300. Mortalin, p53 and BRCA1) and respective β-actin expression presented on Figures S2B and S21. Western blot of protein of interest (p300, PARP ½. Mortalin, p53 and BRCA1) and respective β-actin expression shown on Figure S2C.

## References

[1] Malik F., Kumar A., Bhushan S., Khan S., Bhatia A., Suri K.A., Qazi G.N., Singh J. (2007). Reactive oxygen species generation and mitochondrial dysfunction in the apoptotic cell death of human myeloid leukemia HL-60 cells by a dietary compound withaferin A with concomitant protection by N-acetyl cysteine. Apoptosis.

[2] Stan S.D., Zeng Y., Singh S.V. (2008). Ayurvedic medicine constituent withaferin a causes G2 and M phase cell cycle arrest in human breast cancer cells. Nutr. Cancer.

[3] Choi M.J., Park E.J., Min K.J., Park J.W., Kwon T.K. (2011). Endoplasmic reticulum stress mediates withaferin A-induced apoptosis in human renal carcinoma cells. Toxicol. Vitr..

[4] Lee H.E., Shin J.A., Jeong J.H., Jeon J.G., Lee M.H., Cho S.D. (2016). Anticancer activity of Ashwagandha against human head and neck cancer cell lines. J. Oral Pathol. Med..

[5] Suman S., Das T.P., Sirimulla S., Alatassi H., Ankem M.K., Damodaran C. (2016). Withaferin-A suppress AKT induced tumor growth in colorectal cancer cells. Oncotarget.

[6] Nishikawa Y., Okuzaki D., Fukushima K., Mukai S., Ohno S., Ozaki Y., Yabuta N., Nojima H. (2015). Withaferin A induces cell death selectively in androgen-independent prostate cancer cells but not in normal fibroblast cells. PLoS ONE.

[7] Widodo N., Priyandoko D., Shah N., Wadhwa R., Kaul S.C. (2010). Selective killing of cancer cells by Ashwagandha leaf extract and its component withanone involves ROS signaling. PLoS ONE.

[8] Yu Y., Katiyar S.P., Sundar D., Kaul Z., Miyako E., Zhang Z., Kaul S.C., Reddel R.R., Wadhwa R. (2017). Withaferin-A kills cancer cells with and without telomerase: Chemical, computational and experimental evidences. Cell Death Dis..

[9] Sundar D., Yu Y., Katiyar S.P., Putri J.F., Dhanjal J.K., Wang J., Sari A.N., Kolettas E., Kaul S.C., Wadhwa R. (2019). Wild type p53 function in p53(Y220C) mutant harboring cells by treatment with Ashwagandha derived anticancer withanolides: Bioinformatics and experimental evidence. J. Exp. Clin. Cancer Res..

[10] Bhargava P., Malik V., Liu Y., Ryu J., Kaul S.C., Sundar D., Wadhwa R. (2019). Molecular insights into Withaferin-A-induced senescence: Bioinformatics and experimental evidence to the role of NFkappaB and CARF. J. Gerontol. A Biol. Sci. Med. Sci..

[11] Lahat G., Zhu Q.S., Huang K.L., Wang S., Bolshakov S., Liu J., Torres K., Langley R.R., Lazar A.J., Hung M.C. (2010). Vimentin is a novel anticancer therapeutic target; insights from in vitro and in vivo mice xenograft studies. PLoS ONE.

[12] Thaiparambil J.T., Bender L., Ganesh T., Kline E., Patel P., Liu Y., Tighiouart M., Vertino P.M., Harvey R.D., Garcia A. (2011). Withaferin A inhibits breast cancer invasion and metastasis at sub-cytotoxic doses by inducing vimentin disassembly and serine 56 phosphorylation. Int. J. Cancer.

[13] Mohan R., Bargagna-Mohan P. (2016). The use of Withaferin A to study intermediate filaments. Methods Enzymol..

[14] Grin B., Mahammad S., Wedig T., Cleland M.M., Tsai L., Herrmann H., Goldman R.D. (2012). Withaferin A alters intermediate filament organization, cell shape and behavior. PLoS ONE.

[15] Bargagna-Mohan P., Lei L., Thompson A., Shaw C., Kasahara K., Inagaki M., Mohan R. (2015). Vimentin phosphorylation underlies myofibroblast sensitivity to withaferin a in vitro and during corneal fibrosis. PLoS ONE.

[16] Munagala R., Kausar H., Munjal C., Gupta R.C. (2011). Withaferin a induces p53-dependent apoptosis by repression of HPV oncogenes and upregulation of tumor suppressor proteins in human cervical cancer cells. Carcinogenesis.

[17] Cho M.S., Park W.S., Jung W.K., Qian Z.J., Lee D.S., Choi J.S., Lee D.Y., Park S.G., Seo S.K., Kim H.J. (2014). Caffeic acid phenethyl ester promotes anti-inflammatory effects by inhibiting MAPK and NF-kappaB signaling in activated HMC-1 human mast cells. Pharm. Biol..

[18] Arasoglu T., Derman S., Mansuroglu B. (2016). Comparative evaluation of antibacterial activity of caffeic acid phenethyl ester and PLGA nanoparticle formulation by different methods. Nanotechnology.

[19] Erdemli H.K., Akyol S., Armutcu F., Akyol O. (2015). Antiviral properties of caffeic acid phenethyl ester and its potential application. J. Intercult. Ethnopharmacol..

[20] Wadhwa R., Nigam N., Bhargava P., Dhanjal J.K., Goyal S., Grover A., Sundar D., Ishida Y., Terao K., Kaul S.C. (2016). Molecular characterization and enhancement of anticancer activity of caffeic acid phenethyl ester by gamma cyclodextrin. J. Cancer.

[21] Frenkel K., Wei H., Bhimani R., Ye J., Zadunaisky J.A., Huang M.T., Ferraro T., Conney A.H., Grunberger D. (1993). Inhibition of tumor promoter-mediated processes in mouse skin and bovine lens by caffeic acid phenethyl ester. Cancer Res..

[22] Su Z.Z., Lin J., Grunberger D., Fisher P.B. (1994). Growth suppression and toxicity induced by caffeic acid phenethyl ester (CAPE) in type 5 adenovirus-transformed rat embryo cells correlate directly with transformation progression. Cancer Res..

[23] Jin U.H., Chung T.W., Kang S.K., Suh S.J., Kim J.K., Chung K.H., Gu Y.H., Suzuki I., Kim C.H. (2005). Caffeic acid phenyl ester in propolis is a strong inhibitor of matrix metalloproteinase-9 and invasion inhibitor: Isolation and identification. Clin. Chim. Acta.

[24] Jin U.H., Song K.H., Motomura M., Suzuki I., Gu Y.H., Kang Y.J., Moon T.C., Kim C.H. (2008). Caffeic acid phenethyl ester induces mitochondria-mediated apoptosis in human myeloid leukemia U937 cells. Mol. Cell. Biochem..

[25] Beauregard A.P., Harquail J., Lassalle-Claux G., Belbraouet M., Jean-Francois J., Touaibia M., Robichaud G.A. (2015). CAPE analogs induce growth arrest and apoptosis in breast cancer cells. Molecules.

[26] Firat F., Ozgul M., Turkoz Uluer E., Inan S. (2019). Effects of caffeic acid phenethyl ester (CAPE) on angiogenesis, apoptosis and oxidative stress in various cancer cell lines. Biotech. Histochem..

[27] Chen M.J., Chang W.H., Lin C.C., Liu C.Y., Wang T.E., Chu C.H., Shih S.C., Chen Y.J. (2008). Caffeic acid phenethyl ester induces apoptosis of human pancreatic cancer cells involving caspase and mitochondrial dysfunction. Pancreatology.

[28] Guarini L., Su Z.Z., Zucker S., Lin J., Grunberger D., Fisher P.B. (1992). Growth inhibition and modulation of antigenic phenotype in human melanoma and glioblastoma multiforme cells by caffeic acid phenethyl ester (CAPE). Cell. Mol. Biol..

[29] Ferreira R.S., Dos Santos N.A.G., Martins N.M., Fernandes L.S., Dos Santos A.C. (2018). Caffeic ccid phenethyl ester (CAPE) protects PC12 cells from cisplatin-induced neurotoxicity by activating the NGF-signaling pathway. Neurotox. Res..

[30] Kim H.G., Han E.H., Im J.H., Lee E.J., Jin S.W., Jeong H.G. (2015). Caffeic acid phenethyl ester inhibits 3-MC-induced CYP1A1 expression through induction of hypoxia-inducible factor-1alpha. Biochem. Biophys. Res. Commun..

[31] Lee K.W., Chun K.S., Lee J.S., Kang K.S., Surh Y.J., Lee H.J. (2004). Inhibition of cyclooxygenase-2 expression and restoration of gap junction intercellular communication in H-ras-transformed rat liver epithelial cells by caffeic acid phenethyl ester. Ann. N. Y. Acad. Sci..

[32] Natarajan K., Singh S., Burke T.R., Grunberger D., Aggarwal B.B. (1996). Caffeic acid phenethyl ester is a potent and specific inhibitor of activation of nuclear transcription factor NF-kappa B. Proc. Natl. Acad. Sci. USA.

[33] Song Y.S., Park E.H., Hur G.M., Ryu Y.S., Lee Y.S., Lee J.Y., Kim Y.M., Jin C. (2002). Caffeic acid phenethyl ester inhibits nitric oxide synthase gene expression and enzyme activity. Cancer Lett..

[34] Na H.K., Wilson M.R., Kang K.S., Chang C.C., Grunberger D., Trosko J.E. (2000). Restoration of gap junctional intercellular communication by caffeic acid phenethyl ester (CAPE) in a ras-transformed rat liver epithelial cell line. Cancer Lett..

[35] Messerli S.M., Ahn M.R., Kunimasa K., Yanagihara M., Tatefuji T., Hashimoto K., Mautner V., Uto Y., Hori H., Kumazawa S. (2009). Artepillin c (ARC) in brazilian green propolis selectively blocks oncogenic PAK1 signaling and suppresses the growth of NF tumors in mice. Phytother. Res..

[36] Lee Y.J., Kuo H.C., Chu C.Y., Wang C.J., Lin W.C., Tseng T.H. (2003). Involvement of tumor suppressor protein p53 and p38 MAPK in caffeic acid phenethyl ester-induced apoptosis of C6 glioma cells. Biochem. Pharmacol..

[37] Chuu C.P., Lin H.P., Ciaccio M.F., Kokontis J.M., Hause R.J., Hiipakka R.A., Liao S., Jones R.B. (2012). Caffeic acid phenethyl ester suppresses the proliferation of human prostate cancer cells through inhibition of p70S6K and Akt signaling networks. Cancer Prev. Res. (Phila).

[38] Demestre M., Messerli S.M., Celli N., Shahhossini M., Kluwe L., Mautner V., Maruta H. (2009). CAPE (caffeic acid phenethyl ester)-based propolis extract (Bio 30) suppresses the growth of human neurofibromatosis (NF) tumor xenografts in mice. Phytother. Res..

[39] Wu J., Omene C., Karkoszka J., Bosland M., Eckard J., Klein C.B., Frenkel K. (2011). Caffeic acid phenethyl ester (CAPE), derived from a honeybee product propolis, exhibits a diversity of anti-tumor effects in pre-clinical models of human breast cancer. Cancer Lett..

[40] Liao H.F., Chen Y.Y., Liu J.J., Hsu M.L., Shieh H.J., Liao H.J., Shieh C.J., Shiao M.S., Chen Y.J. (2003). Inhibitory effect of caffeic acid phenethyl ester on angiogenesis, tumor invasion, and metastasis. J. Agric. Food Chem..

[41] Izuta H., Shimazawa M., Tsuruma K., Araki Y., Mishima S., Hara H. (2009). Bee products prevent VEGF-induced angiogenesis in human umbilical vein endothelial cells. BMC Complement Altern. Med..

[42] Chen M.J., Shih S.C., Wang H.Y., Lin C.C., Liu C.Y., Wang T.E., Chu C.H., Chen Y.J. (2013). Caffeic acid phenethyl ester inhibits epithelial-mesenchymal transition of human pancreatic cancer cells. Evid. Based Complement. Altern. Med..

[43] Kuo Y.Y., Lin H.P., Huo C., Su L.C., Yang J., Hsiao P.H., Chiang H.C., Chung C.J., Wang H.D., Chang J.Y. (2013). Caffeic acid phenethyl ester suppresses proliferation and survival of TW2.6 human oral cancer cells via inhibition of Akt signaling. Int. J. Mol. Sci..

[44] Kuo Y.Y., Jim W.T., Su L.C., Chung C.J., Lin C.Y., Huo C., Tseng J.C., Huang S.H., Lai C.J., Chen B.C. (2015). Caffeic Acid phenethyl ester is a potential therapeutic agent for oral cancer. Int. J. Mol. Sci..

[45] Liu C.C., Hsu J.M., Kuo L.K., Chuu C.P. (2013). Caffeic acid phenethyl ester as an adjuvant therapy for advanced prostate cancer. Med. Hypotheses.

[46] Omene C., Kalac M., Wu J., Marchi E., Frenkel K., O’Connor O.A. (2013). Propolis and its active component, caffeic acid phenethyl ester (CAPE), modulate breast cancer therapeutic targets via an epigenetically mediated mechanism of action. J. Cancer Sci. Ther..

[47] Tseng J.C., Lin C.Y., Su L.C., Fu H.H., Yang S.D., Chuu C.P. (2016). CAPE suppresses migration and invasion of prostate cancer cells via activation of non-canonical Wnt signaling. Oncotarget.

[48] Hwang H.J., Park H.J., Chung H.J., Min H.Y., Park E.J., Hong J.Y., Lee S.K. (2006). Inhibitory effects of caffeic acid phenethyl ester on cancer cell metastasis mediated by the down-regulation of matrix metalloproteinase expression in human HT1080 fibrosarcoma cells. J. Nutr. Biochem..

[49] Lee K.W., Kang N.J., Kim J.H., Lee K.M., Lee D.E., Hur H.J., Lee H.J. (2008). Caffeic acid phenethyl ester inhibits invasion and expression of matrix metalloproteinase in SK-Hep1 human hepatocellular carcinoma cells by targeting nuclear factor kappa B. Genes Nutr..

[50] Chen Y.J., Liao H.F., Tsai T.H., Wang S.Y., Shiao M.S. (2005). Caffeic acid phenethyl ester preferentially sensitizes CT26 colorectal adenocarcinoma to ionizing radiation without affecting bone marrow radioresponse. Int. J. Radiat. Oncol. Biol. Phys..

[51] Lee Y.Y., Kao C.L., Tsai P.H., Tsai T.H., Chiou S.H., Wu W.F., Ku H.H., Wong T.T. (2008). Caffeic acid phenethyl ester preferentially enhanced radiosensitizing and increased oxidative stress in medulloblastoma cell line. Childs Nerv. Syst..

[52] Anjaly K., Tiku A.B. (2018). Radio-modulatory potential of caffeic acid phenethyl ester: A therapeutic perspective. Anticancer Agents Med. Chem..

[53] Lee K.J., Choi J.H., Hwang Y.P., Chung Y.C., Jeong H.G. (2008). Protective effect of caffeic acid phenethyl ester on tert-butyl hydroperoxide-induced oxidative hepatotoxicity and DNA damage. Food Chem. Toxicol..

[54] Albukhari A.A., Gashlan H.M., El-Beshbishy H.A., Nagy A.A., Abdel-Naim A.B. (2009). Caffeic acid phenethyl ester protects against tamoxifen-induced hepatotoxicity in rats. Food Chem. Toxicol..

[55] Motawi T.K., Abdelazim S.A., Darwish H.A., Elbaz E.M., Shouman S.A. (2016). Could caffeic acid phenethyl ester expand the antitumor effect of tamoxifen in breast carcinoma?. Nutr. Cancer.

[56] Motawi T.K., Abdelazim S.A., Darwish H.A., Elbaz E.M., Shouman S.A. (2016). Modulation of tamoxifen cytotoxicity by caffeic acid phenethyl ester in MCF-7 breast cancer cells. Oxid. Med. Cell. Longev..

[57] Matsunaga T., Tsuchimura S., Azuma N., Endo S., Ichihara K., Ikari A. (2019). Caffeic acid phenethyl ester potentiates gastric cancer cell sensitivity to doxorubicin and cisplatin by decreasing proteasome function. Anti-Cancer Drugs.

[58] O’Cearbhaill R.E. (2018). Using PARP inhibitors in advanced ovarian cancer. Oncology (Williston Park).

[59] Wu L., Zhong L. (2019). Budget impact analysis of niraparib and olaparib for maintenance treatment of platinum-sensitive, recurrent ovarian cancer in the US. J. Med. Econ..

[60] Putri J.F., Bhargava P., Dhanjal J.K., Yaguchi T., Sundar D., Kaul S.C., Wadhwa R. (2019). Mortaparib, a novel dual inhibitor of mortalin and PARP1, is a potential drug candidate for ovarian and cervical cancers. J. Expt. Clin. Cancer Res..

[61] Vaishnavi K., Saxena N., Shah N., Singh R., Manjunath K., Uthayakumar M. (2012). Differential activities of the two closely related withanolides, withaferin A and withanone: Bioinformatics and experimental evidences. PLoS ONE.

[62] Hegan D.S., Lu Y., Stachelek G.C., Crosby M.E., Bindra R.S., Glazer P.M. (2010). Inhibition of poly(ADP-ribose) polymerase down-regulates BRCA1 and RAD51 in a pathway mediated by E2F4 and p130. Proc. Natl. Acad. Sci. USA.

[63] Mweempwa A., Wilson M.K. (2019). Mechanisms of resistance to PARP inhibitors - an evolving challenge in oncology. Cancer Drug. Resist..

[64] Reilly S.W., Puentes L.N., Wilson K., Hsieh C.J., Weng C.C., Makvandi M., Mach R.H. (2018). Examination of Diazaspiro Cores as Piperazine Bioisosteres in the Olaparib Framework Shows Reduced DNA Damage and Cytotoxicity. J. Med. Chem..

[65] Ridge K.M., Shumaker D., Robert A., Hookway C., Gelfand V.I., Janmey P.A., Lowery J., Guo M., Weitz D.A., Kuczmarski E. (2016). Methods for determining the cellular functions of vimentin intermediate filaments. Methods Enzymol..

[66] Lee D.H., Lim I.H., Sung E.G., Kim J.Y., Song I.H., Park Y.K., Lee T.J. (2013). Withaferin A inhibits matrix metalloproteinase-9 activity by suppressing the Akt signaling pathway. Oncol. Rep..

[67] Um H.J., Min K.J., Kim D.E., Kwon T.K. (2012). Withaferin A inhibits JAK/STAT3 signaling and induces apoptosis of human renal carcinoma Caki cells. Biochem. Biophys. Res. Commun..

[68] Khan S., Khamis I., Heikkila J.J. (2015). The small heat shock protein, HSP30, is associated with aggresome-like inclusion bodies in proteasomal inhibitor-, arsenite-, and cadmium-treated Xenopus kidney cells. Comp. Biochem. Physiol. A Mol. Integr. Physiol..

[69] Sharda A.C., Solomon F.E., Devi P.U., Udupa N., Srinivasan K.K. (1996). Antitumor and radisensitizing effects of withaferin a on mous EHRLICH ascites carcinoma In vivo. Acta Oncol..

[70] Pires N., Gota V., Gulia A., Hingorani L., Agarwal M., Puri A. (2020). Safety and pharmacokinetics of withaferin-a in advanced stage high grade osteosarcoma: A phase I trial. J. Ayurveda Integr. Med..

[71] Gao R., Shah N., Lee J.S., Katiyar S.P., Li L., Oh E., Sundar D., Yun C.O., Wadhwa R., Kaul S.C. (2014). Withanone-rich combination of Ashwagandha withanolides restricts metastasis and angiogenesis through hnRNP-K. Mol. Cancer Ther..

[72] Kakar S.S., Ratajczak M.Z., Powell K.S., Moghadamfalahi M., Miller D.M., Batra S.K., Singh S.K. (2014). Withaferin a alone and in combination with cisplatin suppresses growth and metastasis of ovarian cancer by targeting putative cancer stem cells. PLoS ONE.

[73] Garg S., Huifu H., Kumari A., Sundar D., Kaul S.C., Wadhwa R. (2019). Induction of senescence in cancer cells by a novel combination of cucurbitacon B and withanone: Molecular mechanism and therapeutic potential. J. Gerontol. A Biol. Sci. Med. Sci..

[74] Jung B.I., Kim M.S., Kim H.A., Kim D., Yang J., Her S., Song Y.S. (2010). Caffeic acid phenethyl ester, a component of beehive propolis, is a novel selective estrogen receptor modulator. Phytother Res..

[75] Ishida Y., Gao R., Shah N., Bhargava P., Furune T., Kaul S.C., Terao K., Wadhwa R. (2018). Anticancer activity in honeybee propolis: Functional insights to the role of caffeic acid phenethyl ester and its complex with gamma-cyclodextrin. Integr. Cancer Ther..

[76] Hsu T.H., Chu C.C., Hung M.W., Lee H.J., Hsu H.J., Chang T.C. (2013). Caffeic acid phenethyl ester induces E2F-1-mediated growth inhibition and cell-cycle arrest in human cervical cancer cells. FEBS J..

[77] Bhargava P., Kumari A., Putri J.F., Ishida Y., Terao K., Kaul S.C., Sundar D., Wadhwa R. (2018). Caffeic acid phenethyl ester (CAPE) possesses pro-hypoxia and anti-stress activities: Bioinformatics and experimental evidences. Cell Stress Chaperones.

[78] Tolba M.F., Esmat A., Al-Abd A.M., Azab S.S., Khalifa A.E., Mosli H.A., Abdel-Rahman S.Z., Abdel-Naim A.B. (2013). Caffeic acid phenethyl ester synergistically enhances docetaxel and paclitaxel cytotoxicity in prostate cancer cells. IUBMB Life.

[79] Widodo N., Kaur K., Shrestha B.G., Takagi Y., Ishii T., Wadhwa R., Kaul S.C. (2007). Selective killing of cancer cells by leaf extract of Ashwagandha: Identification of a tumor-inhibitory factor and the first molecular insights to its effect. Clin. Cancer Res..

[80] Lu W.J., Lee N.P., Kaul S.C., Lan F., Poon R.T., Wadhwa R., Luk J.M. (2011). Mortalin-p53 interaction in cancer cells is stress dependent and constitutes a selective target for cancer therapy. Cell Death Differ..

[81] Lu W.J., Lee N.P., Kaul S.C., Lan F., Poon R.T., Wadhwa R., Luk J.M. (2011). Induction of mutant p53-dependent apoptosis in human hepatocellular carcinoma by targeting stress protein mortalin. Int. J. Cancer.

[82] Flachbartova Z., Kovacech B. (2013). Mortalin—A multipotent chaperone regulating cellular processes ranging from viral infection to neurodegeneration. Acta Virol..

[83] Scheffner M., Werness B.A., Huibregtse J.M., Levine A.J., Howley P.M. (1990). The E6 oncoprotein encoded by human papillomavirus types 16 and 18 promotes the degradation of p53. Cell.

[84] Kostecka A., Sznarkowska A., Meller K., Acedo P., Shi Y., Sakil H.M., Kawiak A., Lion M., Królicka A., Wilhelm M. (2014). JNK–NQO1 axis drives TAp73-mediated tumor suppression upon oxidative and proteasomal stress. Cell Death Dis..

[85] Wadhwa R., Priyandoko D., Gao R., Widodo N., Nigam N., Li L., Ahn H.M., Yun C.O., Ando N., Mahe C. (2016). Stress chaperone mortalin regulates human melanogenesis. Cell Stress Chaperones.

[86] Lourenco L.M., Jiang Y., Drobnitzky N., Green M., Cahill F., Patel A., Shanneik Y., Moore J., Ryan A.J. (2018). PARP inhibition combined with thoracic irradiation exacerbates esophageal and skin toxicity in C57BL6 mice. Int. J. Radiat. Oncol. Biol. Phys..

[87] Fang P., Madden J.A., Neums L., Moulder R.K., Forrest M.L., Chien J. (2018). Olaparib-induced adaptive response is disrupted by FOXM1 targeting that enhances sensitivity to PARP inhibition. Mol. Cancer Res..

[88] Griguolo G., Dieci M.V., Guarneri V., Conte P. (2018). Olaparib for the treatment of breast cancer. Expert Rev. Anticancer Ther..

[89] Wang X., Shi Y., Huang D., Guan X. (2018). Emerging therapeutic modalities of PARP inhibitors in breast cancer. Cancer Treat. Rev..

[90] Wu M., Liu J., Hu C., Li D., Yang J., Wu Z., Yang L., Chen Y., Fu S., Wu J. (2018). Olaparib nanoparticles potentiated radiosensitization effects on lung cancer. Int. J. Nanomed..

[91] Vel Szic K.S., De Beeck K.O., Ratman D., Wouters A., Beck I.M., Declerck K., Heyninck K., Fransen E., Bracke M., De Bosscher K. (2014). Pharmacological levels of withaferin A (*Withania somnifera*) trigger clinically relevant anticancer effects specific to triple negative breast cancer cells. PLoS ONE.

[92] Oben K.Z., Alhakeem S.S., McKenna M.K., Brandon J.A., Mani R., Noothi S.K., Jinpeng L., Akunuru S., Dhar S.K., Singh I.P. (2017). Oxidative stress-induced JNK/AP-1 signaling is a major pathway involved in selective apoptosis of myelodysplastic syndrome cells by Withaferin-A. Oncotarget.

[93] Khan S., Rammeloo A.W., Heikkila J.J. (2012). Withaferin-A induces proteasome inhibition, endoplasmic reticulum stress, the heat shock response and acquisition of thermotolerance. PLoS ONE.

[94] Chou T.C., Talalay P. (1984). Quantitative analysis of dose-effect relationships: The combined effects of multiple drugs or enzyme inhibitors. Adv. Enzym. Regul..

[95] (2020). Schrödinger Release 2020-1: Maestro 019-3 SR, Glide, LigPrep, ProteinPreparation Wizard, Prime, Molecular Dynamics System.

[96] Dawicki-McKenna J.M., Langelier M.F., DeNizio J.E., Riccio A.A., Cao C.D., Karch K.R., McCauley M., Steffen J.D., Black B.E., Pascal J.M. (2015). PARP1 activation requires local unfolding of an autoinhibitory domain. Mol. Cell..

