# Supplementary material for: Combination of Withaferin-A and CAPE Provides Superior Anticancer Potency: Bioinformatics and Experimental Evidence to Their Molecular Targets and Mechanism of Action
Source: Cancers (Basel). 2020 May 5;12(5):1160. doi: 10.3390/cancers12051160 (PMC7281427; doi:10.3390/cancers12051160)
Supplement: Supplementary file 1 [file cancers-12-01160-s001.pdf]

## Supplementary Materials

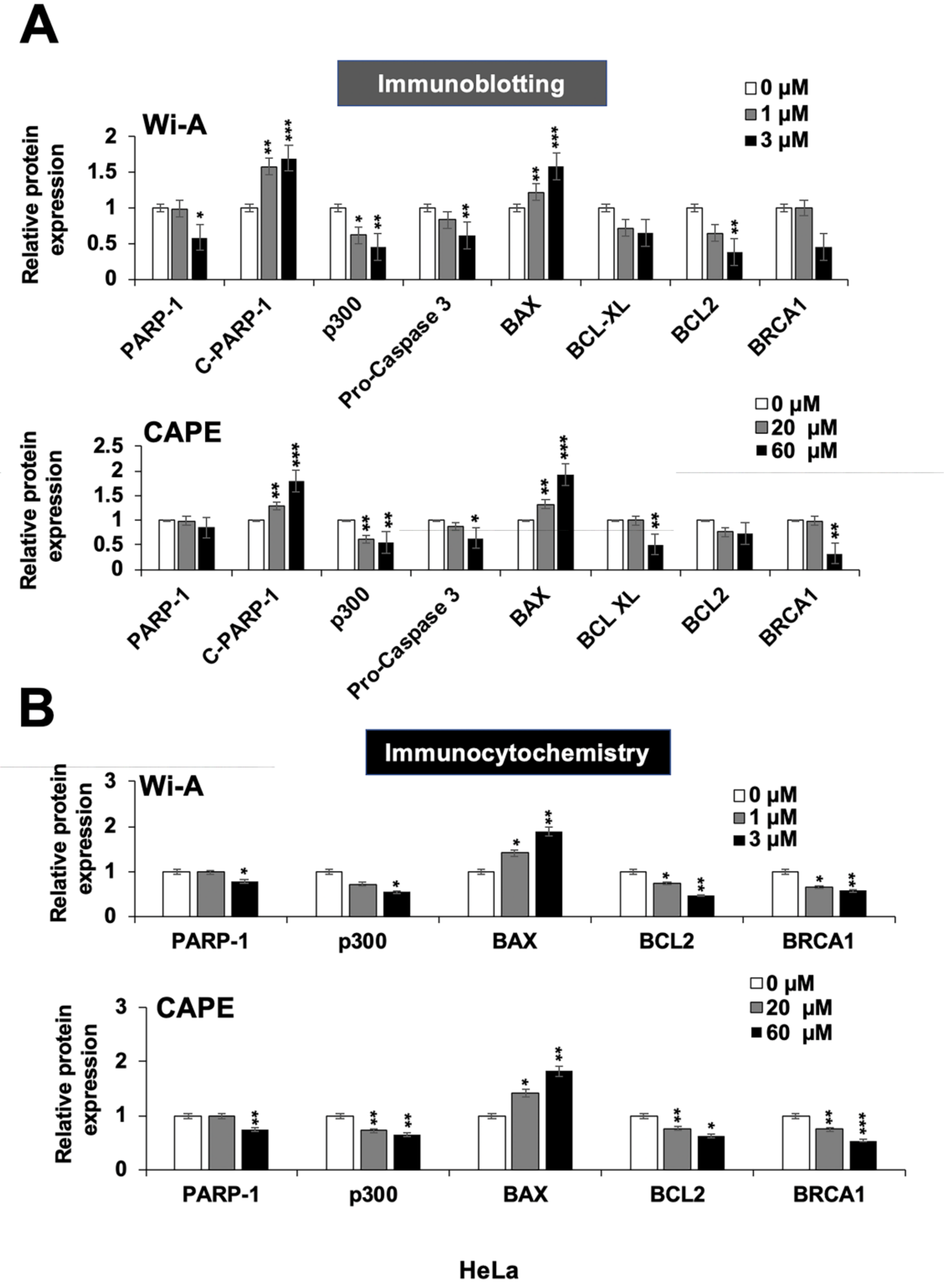


**Figure S1.** Quantitation of apoptosis markers modulated by Wi-A and CAPE treatment (**A**) Western blotting (**B**) Immunostaining**.** Data were normalized against control and plotted as fold difference. Each data set represents the mean ± SD for at least 3 independent biological replicates. Statistical significance was defined as *p*-values (*) where * < 0.05, ** < 0.01 and *** < 0.001 represent significant, very significant and highly significant, respectively.

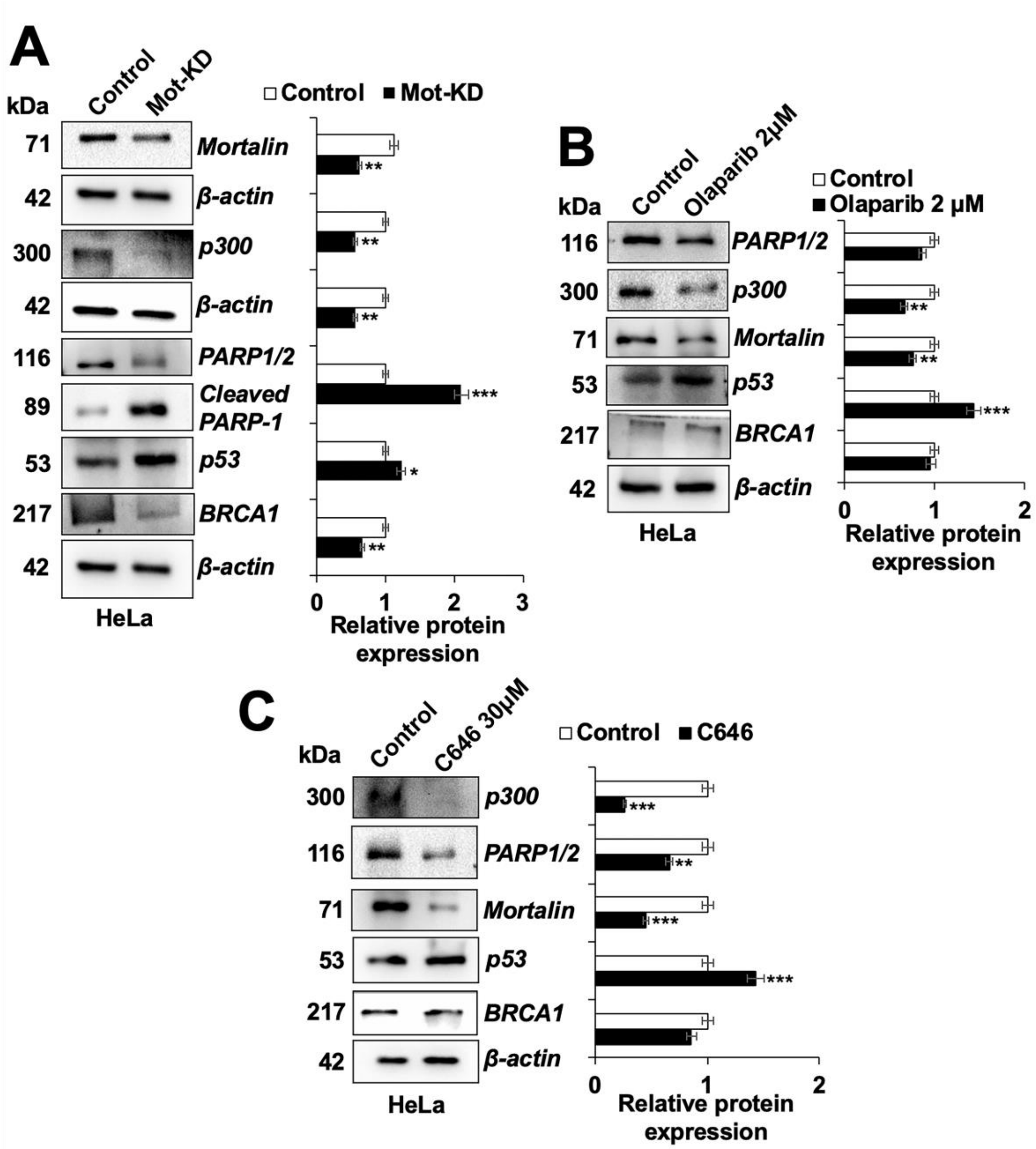


**Figure S2.** Effect of mortalin on PARP1 and *vice versa*. Mortalin-compromised cells showed decrease in PARP1, p300, BRCA1 (**A**). Olaparib (PARP1 inhibitor) and C646 (P300 inhibitor) caused decrease in mortalin (**B**,**C**). Data were normalized against control and plotted as fold difference. Each data set represents the mean ± SD for at least 3 independent biological replicates. Statistical significance was defined as *p*-values (*) where * < 0.05, ** < 0.01 and *** < 0.001 represent significant, very significant and highly significant, respectively.

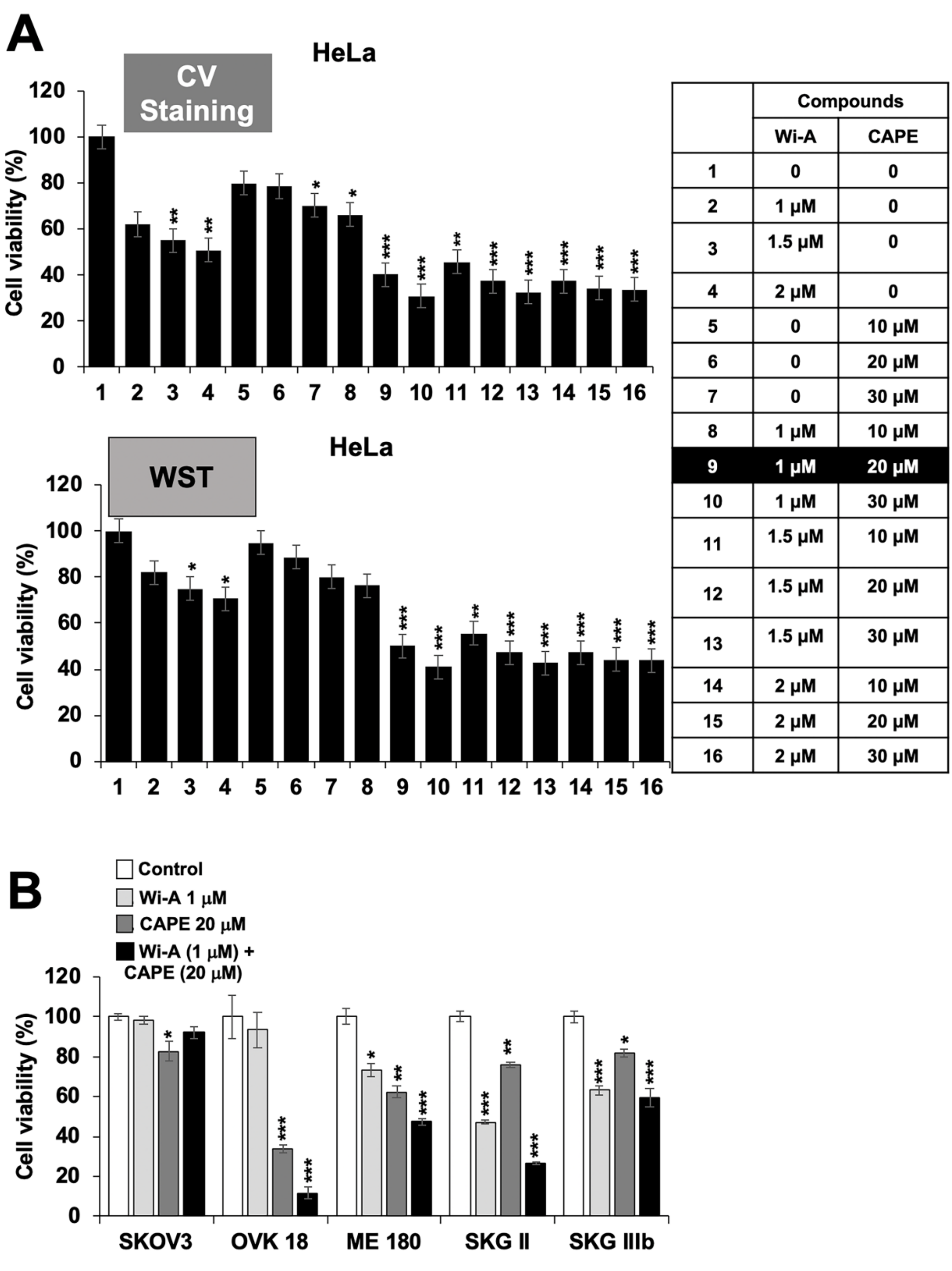


| | Compounds | |
|---|---|---|
| | Wi-A | CAPE |
| 1 | 0 | 0 |
| 2 | 1 μM | 0 |
| 3 | 1.5 μM | 0 |
| 4 | 2 μM | 0 |
| 5 | 0 | 10 μM |
| 6 | 0 | 20 μM |
| 7 | 0 | 30 μM |
| 8 | 1 μM | 10 μM |
| 9 | 1 μM | 20 μM |
| 10 | 1 μM | 30 μM |
| 11 | 1.5 μM | 10 μM |
| 12 | 1.5 μM | 20 μM |
| 13 | 1.5 μM | 30 μM |
| 14 | 2 μM | 10 μM |
| 15 | 2 μM | 20 μM |
| 16 | 2 μM | 30 μM |

**Figure S3.** WST and CV staining-based cell viability assay showing dose dependent cytotoxicity of either Wi-A or CAPE or their combination in HeLa cells **(A).** Cytotoxicity of Wi-A, CAPE and their combination to different ovarian and cervical cancer cells **(B).** Data were normalized against control and plotted as percent difference. Each data set represents the mean ± SD for at least 3 independent biological replicates. Statistical significance was defined as *p*-values (*) where * < 0.05, ** < 0.01 and *** < 0.001 represent significant, very significant and highly significant, respectively.

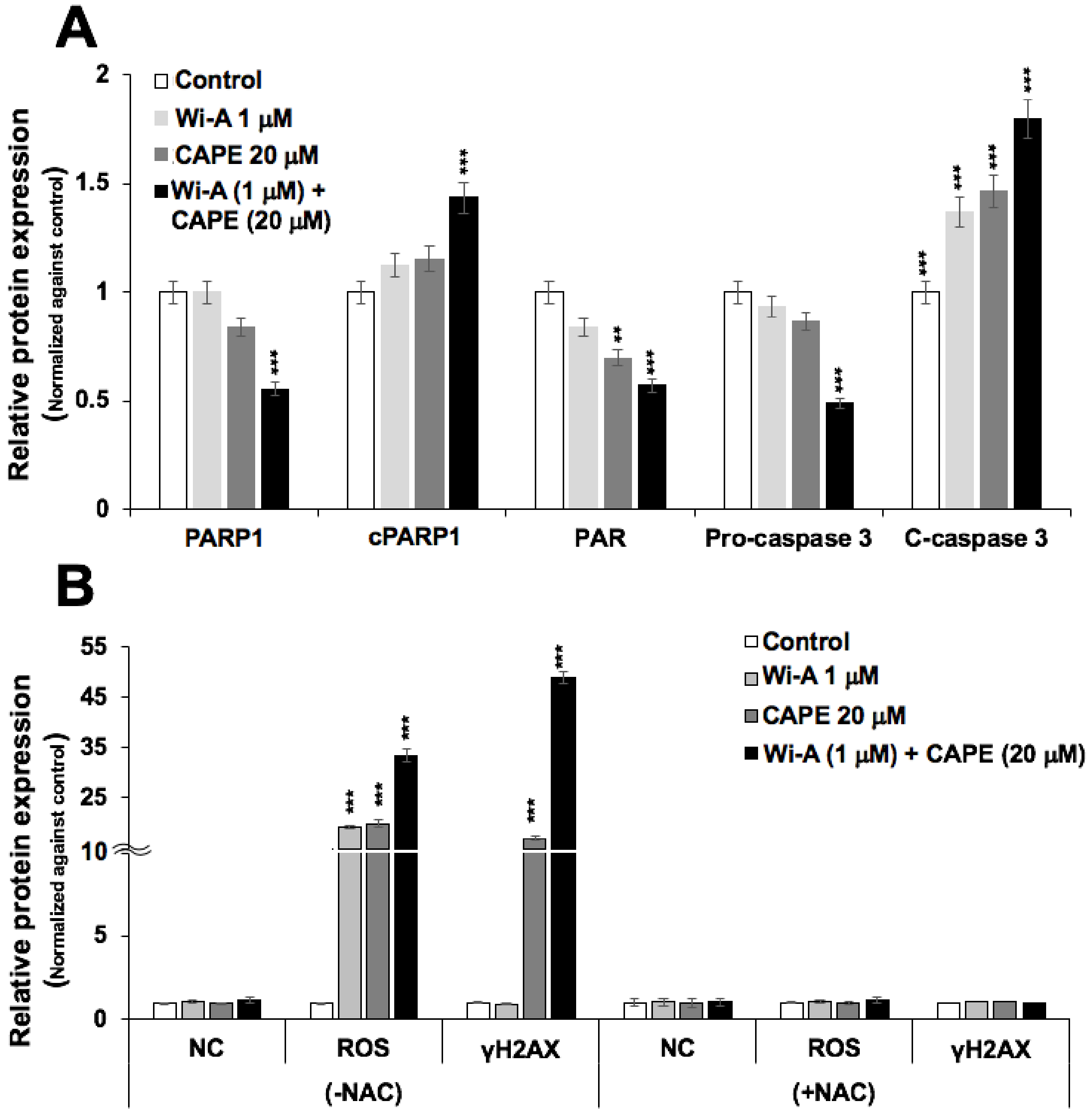


**Figure S4.** (**A**) Quantitation of apoptotic markers in control and treated (Wi-A, CAPE and Combination) cells from the data shown in Figure 5A. (**B**) Quantitative measurement of fluorescence intensity of ROS and γH2AX in control and treated (Wi-A, CAPE and Combination) cells, in the presence/absence of NAC from the data presented in Figure 6C. Data were normalized against the control, and plotted as fold difference. Data set represents the mean ± SD for at least 3 independent biological replicates. Statistical significance was defined as *p*-values where ** < 0.01 and *** < 0.001 represent very significant and highly significant, respectively.

**A**

| | | Wi-A | CAPE |
|---|---|---|---|
| p53 | Mortalin-binding region: 323-337 | Stable interaction<br>Docking score: -2.008 | No stable interaction |
| Mortalin | ATPase domain<br>(p53 binding region: 253-282) | No stable interaction | Stable interaction<br>Docking score: -2.396 |
| | Substrate binding domain<br>(Latch-1: 477, 513, 586, 590) | Stable interaction<br>Docking score: -1.975 | Stable interaction<br>Docking score: -6.949 |
| | Substrate binding domain<br>(Latch-2: 574, 578, 628) | Stable interaction<br>Docking score: -3.036 | No stable interaction |

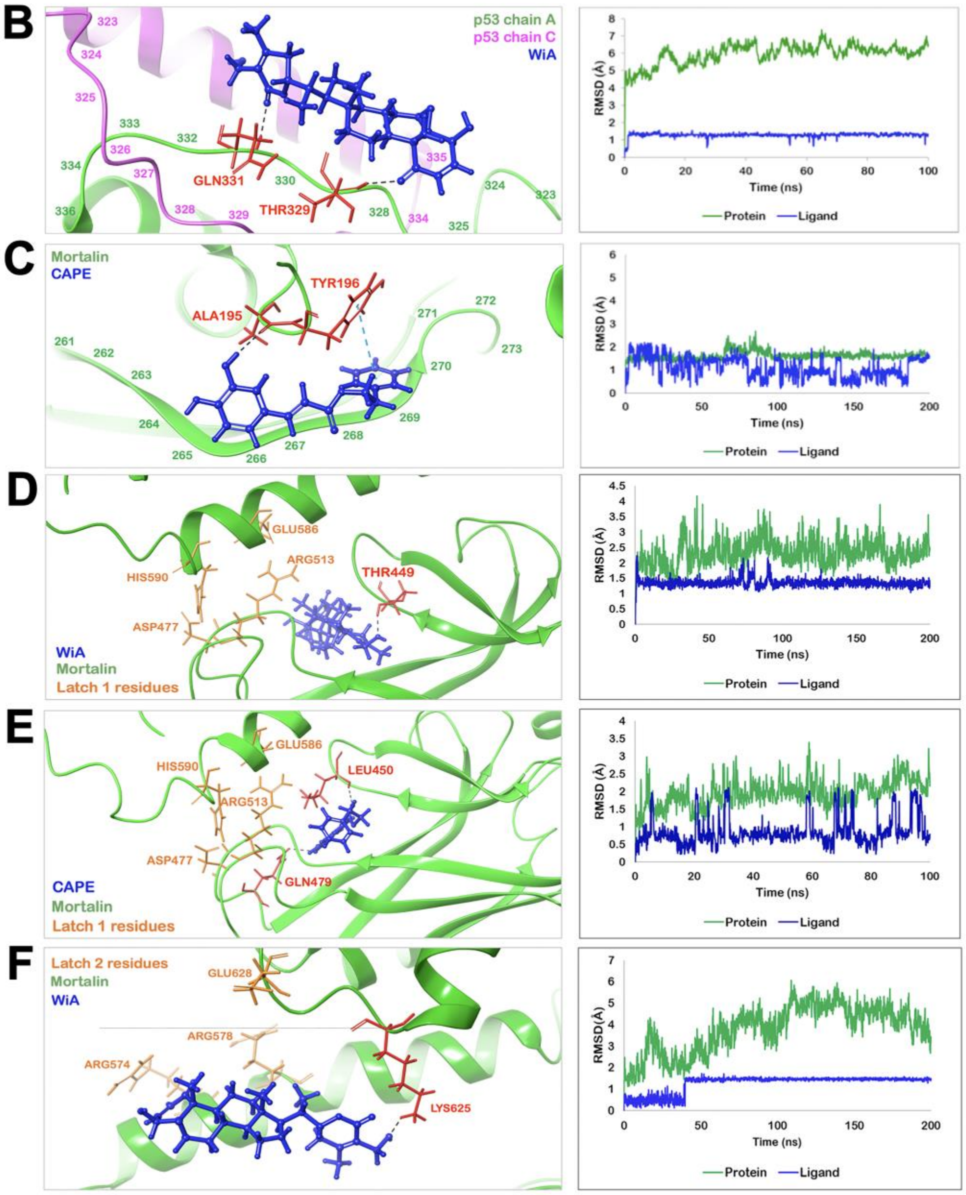


**Figure S5.** Molecular dockings and simulations to study the interaction of Wi-A and CAPE to target proteins, mortalin and p53. (**A**) Summary of interactions between protein-ligand complexes. (**B**) Interaction of Wi-A in mortalin binding site (323-337) of p53. (**C**) Interaction of CAPE in p53 binding site (253-282) of mortalin. (**D**) Interaction of Wi-A with latch 1 in substrate binding pocket of mortalin. (**E**) Interaction of CAPE with latch 1 in substrate binding pocket of mortalin. (**F**) Interaction of Wi-A with latch 2 in substrate binding pocket of mortalin. .
